# Improving prescribing practices with rapid diagnostic tests (RDTs): synthesis of 10 studies to explore reasons for variation in malaria RDT uptake and adherence

**DOI:** 10.1136/bmjopen-2016-012973

**Published:** 2017-03-08

**Authors:** Helen E D Burchett, Baptiste Leurent, Frank Baiden, Kimberly Baltzell, Anders Björkman, Katia Bruxvoort, Siân Clarke, Deborah DiLiberto, Kristina Elfving, Catherine Goodman, Heidi Hopkins, Sham Lal, Marco Liverani, Pascal Magnussen, Andreas Mårtensson, Wilfred Mbacham, Anthony Mbonye, Obinna Onwujekwe, Denise Roth Allen, Delér Shakely, Sarah Staedke, Lasse S Vestergaard, Christopher J M Whitty, Virginia Wiseman, Clare I R Chandler

**Affiliations:** 1 Department of Global Health and Development, London School of Hygiene and Tropical Medicine, London, UK; 2 Department of Infectious Disease Epidemiology, London School of Hygiene and Tropical Medicine, London, UK; 3 Epidemiology Unit, Ensign College of Public Health, Kpong, Ghana; 4 Department of Family Health Care Nursing, and Global Health Science, University of California, Berkeley, California, USA; 5 Department of Microbiology, Tumour and Cell Biology, Karolinska Institute, Stockholm, Sweden; 6 Disease Control Department, London School of Hygiene and Tropical Medicine, London, UK; 7 Clinical Research Department, London School of Hygiene and Tropical Medicine, London, UK; 8 Department of Infectious Diseases, Sahlgrenska Academy, University of Gothenburg, Goteborg, Sweden; 9 Department of Paediatrics, Sahlgrenska Academy, University of Gothenburg, Goteborg, Sweden; 10 Department of Microbiology, Tumour and Cell Biology, Karolinska Institutet, Stockholm, Sweden; 11 Faculty of Health and Medical Sciences, Centre for Medical Parasitology, University of Copenhagen, Copenhagen, Denmark; 12 Department of Women's and Children's Health, Uppsala University, Uppsala, Sweden; 13 Laboratory for Public Health Research Biotechnologies, The Biotechnology Center, University of Yaoundé, Yaoundé, Cameroon; 14 School of Public Health- Makerere University and Commissioner Health Services, Ministry of Health, Uganda; 15 Department of Pharmacology and Therapeutics, University of Nigeria Enugu-Campus, Nigeria; 16 Centers for Disease Control and Prevention (CDC), USA; 17 Department of Medicine, Kungälv Hospital, Sweden; 18 Centre for Medical Parasitology, University of Copenhagen and Copenhagen University Hospital Rigshospitalet, Denmark; 19 Department of Infectious Disease Epidemiology, Statens Serum Institut, Denmark; 20 School of Public Health and Community Medicine, Australia

**Keywords:** TROPICAL MEDICINE

## Abstract

**Objectives:**

The overuse of antimalarial drugs is widespread. Effective methods to improve prescribing practice remain unclear. We evaluated the impact of 10 interventions that introduced rapid diagnostic tests for malaria (mRDTs) on the use of tests and adherence to results in different contexts.

**Design:**

A comparative case study approach, analysing variation in outcomes across different settings.

**Setting:**

Studies from the ACT Consortium evaluating mRDTs with a range of supporting interventions in 6 malaria endemic countries. Providers were governmental or non-governmental healthcare workers, private retail sector workers or community volunteers. Each study arm in a distinct setting was considered a case.

**Participants:**

28 cases from 10 studies were included, representing 148 461 patients seeking care for suspected malaria.

**Interventions:**

The interventions included different mRDT training packages, supervision, supplies and community sensitisation.

**Outcome measures:**

Analysis explored variation in: (1) uptake of mRDTs (% febrile patients tested); (2) provider adherence to positive mRDTs (% *Plasmodium falciparum* positive prescribed/given Artemisinin Combination Treatment); (3) provider adherence to negative mRDTs (% *P. falciparum* negative not prescribed/given antimalarial).

**Results:**

Outcomes varied widely across cases: 12–100% mRDT uptake; 44–98% adherence to positive mRDTs; 27–100% adherence to negative mRDTs. Providers appeared more motivated to perform well when mRDTs and intervention characteristics fitted with their own priorities. Goodness of fit of mRDTs with existing consultation and diagnostic practices appeared crucial to maximising the impact of mRDTs on care, as did prior familiarity with malaria testing; adequate human resources and supplies; possible alternative treatments for mRDT-negative patients; a more directive intervention approach and local preferences for ACTs.

**Conclusions:**

Basic training and resources are essential but insufficient to maximise the potential of mRDTs in many contexts. Programme design should respond to assessments of provider priorities, expectations and capacities. As mRDTs become established, the intensity of supporting interventions required seems likely to reduce.

**Strengths and limitations of this study**

- This analysis addresses the gap in knowledge around how to change prescribing practices, a key question in the era of resistance to antimicrobial medicines.
- The analysis exploits indepth data from 10 intervention studies connected through the ACT Consortium in order to explore the reasons for variation in trial outcomes.
- A comparative case study approach was used, allowing trends and patterns to be explored across contexts in a way not possible within single studies.
- By analysing studies conducted within a consortium, access to unpublished documents, raw data and qualitative insights from the study teams allowed a deeper understanding of the studies and their contexts than is often found in systematic reviews of published reports.
- The extent of variation across the study arms in terms of context, provider type, intervention content and study design allowed for exploration of a range of factors affecting outcomes, but also created challenges for comparability, necessitating a case study approach.

## Background

The substantial overdiagnosis of malaria as a cause of acute febrile illness has been the focus of global attention in recent years,^1–3^ given concerns about the clinical effects of misdiagnoses, the cost of first-line artemisinin-based combination therapies (ACTs) and emerging malaria drug resistance.^4^ ^5^ A policy of universal parasitological testing for malaria was introduced by the WHO in 2010,^6^ aiming to reduce overprescription of ACTs.^2^ Malaria rapid diagnostic tests (mRDTs) have been developed for use in low-resource settings, making parasite-based testing possible where microscopy may not be available or feasible.^4^

RDTs have been introduced with providers in a range of sectors.^7^ However, evidence from evaluations of mRDT introductions show mixed effects; mRDTs do not lead to improved targeting of ACTs if providers do not consistently use the tests or if they ignore test results.^8–12^ To maximise their potential for improving prescribing practices, evidence is required of the relative success and challenges of different types of mRDT intervention in different contexts.

This paper presents an analysis of the findings from 10 mRDT intervention studies conducted in Africa and Afghanistan, for which indepth information was available about interventions, outcomes and contexts. The studies, all from the ACT Consortium, represent a large proportion of the intervention studies on mRDTs recently conducted in areas of ongoing malaria transmission. This analysis aimed to identify how mRDTs can be used to improve prescribing in different contexts by exploring factors influencing providers' use of and adherence to test results and comparing results of interventions in different settings.

## Methods

The ACT Consortium is an international research collaboration involving more than 20 institutions working on a systematic series of 25 studies in 10 countries in Africa and Asia, addressing practical questions in the delivery of malaria treatment.^13^ Intervention studies involving mRDTs were conducted in 10 sites in 6 countries. The analysis in this paper focuses on these studies because of the ability it gives to use raw outcome data (allowing comparable outcomes to be calculated), raw data from linked qualitative research, unpublished documentation about intervention content, implementation and contextual information as well as insights from the study teams. This allowed a more detailed and comparable analysis than could be achieved through reliance on publications or quantitative data alone.

This analysis used a comparative case study approach, where each study arm conducted in a distinct setting was considered a case and outcomes were interpreted in terms of the study design, intervention content, implementation and contextual factors.^14^ This approach suits investigation of ‘how’ and ‘why’ interventions have an effect and can highlight comparative general trends and distinct patterns that are not visible in single cases.^15^ ^17^ The analysis explored three outcomes:

1. Provider uptake of mRDTs.
  The proportion of patients presenting with fever, or history of fever in past 48 hours (unless specified otherwise), who were tested for malaria with an mRDT, as reported by the provider or patient.
2. Provider adherence to positive mRDT results.
  The proportion of patients with a positive mRDT result (for *Plasmodium falciparum* malaria), who were prescribed or received an ACT, the first-line drug for non-severe malaria in all cases, as reported by provider or patient.
3. Provider adherence to negative mRDT results.
  The proportion of patients with a negative mRDT result who were *not* prescribed, or did *not* receive, any antimalarial as reported by provider or patient (the effect of negative mRDT results on the use of other treatments, including antibiotics, in ACT Consortium studies has been presented in a separate paper).^16^

The analysis evaluated the impact of different interventions to introduce mRDTs in different contexts. Twenty-eight cases (ie, distinct settings or intervention arms) from the 10 studies were included, with a total of 148 461 patients (see table 1). Twenty cases from 7 studies analysed mRDT uptake, 24 cases from 9 studies evaluated provider adherence to positive mRDT results and all 28 cases analysed provider adherence to negative mRDT results.

**Table 1:** Cases included in analysis

Study	Study name	Country	Providers targeted	Cases*	Published results
Afgh1	Strategies for expanding access to quality malaria diagnosis in south-central Asia where malaria incidence is low	Afghanistan	Government primary care providers	Afgh1/a: training; patients individually randomised to receive either mRDT or established microscopy, Eastern province	18–20
				Afgh1/b: training; patients individually randomised to receive either mRDT or recently introduced microscopy, Northern province	
				Afgh1/c: training; patients individually randomised to receive either mRDT or clinical diagnosis (no microscopy available), Northern province	
Cam1	Cost-effectiveness of interventions to support the introduction of malaria rapid diagnostic tests in Cameroon	Cameroon	Government and mission providers (in hospitals and primary care)	Cam1/a1: basic training, Bamenda	21–27
				Cam1/b1: basic training, Yaoundé	
				Cam1/a2: enhanced training, Bamenda	
				Cam1/b2: enhanced training, Yaoundé	
Ghan1	How the use of rapid diagnostic tests influences clinicians' decision to prescribe ACTs	Ghana	Government primary care providers	Ghan1/a: training; patients individually randomised to receive either mRDT or microscopy	28–30
			Government and private primary care providers	Ghan1/b: training; patients individually randomised to receive either mRDT or clinical diagnosis	
Nig1	Costs and effects of strategies to improve malaria diagnosis and treatment in Nigeria	Nigeria	Government primary care providers, private pharmacies and private medicine dealers	Nig1/a1: basic training, Enugu	27 31–34
				Nig1/b1: basic training, Udi	
				Nig1/a2: enhanced training, Enugu	
				Nig1/b2: enhanced training, Udi	
				Nig1/a3: enhanced training + school activities, Enugu	
				Nig1/b3: enhanced training + school activities, Udi	
Tanz1	IMPACT 2: Evaluating policies in Tanzania to improve malaria diagnosis and treatment	Tanzania	Government healthcare providers (in hospitals and primary care)	Tanz1/a: standard MoH† training, Mwanza, moderate transmission	35
				Tanz1/b: standard MoH training, Mbeya, low transmission	
				Tanz1/c: standard MoH training, Mtwara, moderate transmission	
Tanz2	Targeting ACT drugs: the TACT trial	Tanzania	Government primary care providers	Tanz2/a1: pilot study, low transmission	36–38
				Tanz2/b1: pilot study, moderate transmission	
				Tanz2/2: basic training	
				Tanz2/3: enhanced training	
				Tanz2/4: enhanced training + patient sensitisation	
Tanz3	Effectiveness of malaria rapid diagnostic tests in fever patients attending primary healthcare facilities in Zanzibar	Tanzania	Government primary care providers	Tanz3: enhanced training, Zanzibar	39 40
Uga1	The PRIME trial: improving health centres to reduce childhood malaria in Uganda	Uganda	Government primary healthcare providers	Uga1: training, Tororo	41–44 72 73
Uga2	Use of rapid diagnostic tests to improve malaria treatment in the community in Uganda	Uganda	Community health volunteers	Uga2/a: training, low transmission	45 74
				Uga2/b: training, moderate transmission	
Uga3	Introducing rapid diagnostic tests in drug shops to improve the targeting of malaria treatment	Uganda	Private drug shop vendors	Uga3: training, Mukono	46–51

*The initial letters refer to the study country, the first number refers to the (country-specific) study number, the subsequent letter refers to the specific context if a study took place in multiple geographical or epidemiological settings and the final number refers to the intervention arm.

†MoH, Ministry of Health.

The studies took place between 2007 and 2012. Studies were either individual (n=2) or cluster-randomised controlled trials (n=6); observational (n=2) or preintervention/postintervention studies (n=1) (Tanz2 used different designs in their pilot and main study, so n=11). Providers targeted were governmental or non-governmental healthcare workers, private retail sector workers or community health volunteers. Six studies took place in East Africa, three in West Africa^Cam1,Nig1,Ghan1^ and one in south-central Asia^Afgh1^. One focused only on children under 5 years^Uga2^; the rest included children and adults. See online supplementary file 1 for more detailed information about each study.

10.1136/bmjopen-2016-012973.supp1 supplementary file

All the interventions included basic training on malaria testing with RDTs for healthcare providers, however the content, duration and approach varied. Some interventions included additional activities and materials such as extra training, supervision and feedback, patient information leaflets or school-based activities (see table 2 and online supplementary file 1).

**Table 2:** Intervention content

Scenario	mRDT/malaria training	Supervision	mRDT/ACT supplies	Other intervention activities
Afgh1/a	One and a half day training, following the national training package. This covered performing mRDTs (most, but not all, practiced testing) and prescribing antimalarials	None	mRDTs supplied by study	None
Afgh1/b				
Afgh1/c				
Cam1/a1	One day, didactic session covered three modules: malaria diagnosis, mRDTs, and malaria treatment	Monthly	mRDTs and ACTs supplied by study	None
Cam1/b1				
Cam1/a2	Same as Cam1/1, plus: Interactive two day training on adapting to change (focused on WHO malaria treatment guidelines), professionalism and effective communication	Monthly	mRDTs and ACTs supplied by study	None
Cam1/b2				
Ghan1/a	Two day training about the sensitivity and specificity of mRDTs, alternative causes of febrile illness and the Ghana national guidelines (which indicated presumptive treatment for children who are <5 years old)	None, but study team were present	mRDTs supplied by study	None
Ghan1/b				
Nig1/a1	Half day demonstration on how to use mRDTs, which included practising conducting one test. They also received a copy of the WHO job aid, which shows the steps in using an mRDT	None	mRDTs supplied by study	None
Nig1/b1				
Nig1/a2	Same as Nig1/1, plus: Two day interactive, seminar-style training, covering how to test, appropriate treatment for positive and negative results and effective communication. Those attending were given job aids (eg, treatment algorithm)	Monthly	mRDTs supplied by study	None
Nig1/b2				
Nig1/a3	Same as Nig1/2	Monthly	mRDTs supplied by study	School-based activities
Nig1/b3				
Tanz1/a	Two day training (standard MoH), covering performing mRDTs (including practical) and prescribing antimalarials	Routine MoH supervision only	mRDTs supplied by MoH	None
Tanz1/b				
Tanz1/c				
Tanz2/a1	One day training on how to use the mRDT and read the result. Antimalarial drug use guidelines were reviewed and job aids provided	None	mRDTs supplied by study	None
Tanz2/b1				
Tanz2/2	Two day, didactic, MoH training on how to use mRDTs, including practical	Six-weekly, focused on supplies and reporting	mRDTs supplied by study	None
Tanz2/3	Same as Tanz2/2, plus: Three additional 90 min interactive training workshops, with one session repeated 6–7 months later. These covered: adapting to the change in the diagnosis and management of malaria; practice with confidence when using mRDTs: tools to enable change in managing febrile illness; sustaining the change in practice. Training on communication skills was included	Six-weekly, focused on supplies and reporting	mRDTs supplied by study	SMS feedback on own mRDT uptake and adherence at 5 months Two times per day motivational SMS for 15 days
Tanz2/4	Same as Tanz2/3	Six-weekly, focused on supplies and reporting	mRDTs supplied by study	SMS feedback on own mRDT uptake and adherence at 5 months Two times per day motivational SMS for 15 days. Patient leaflets and posters
Tanz3	Six to 11 days IMCI training (depending on whether refresher training or for new health workers) which included malaria diagnosis and treatment, plus 1-week study-specific training (including good clinical practice, provision of informed consent, performance and interpretation of mRDT according to the manufacturer's instructions). One day of the IMCI training focused specifically on malaria. Training covered communication skills	None	mRDTs and ACTs supplied by MoH, with study back up in the case of stockouts	IMCI training, additional study salary for providers
Uga1	Two day training session followed a week later by on-site training in facilities. Training was interactive and included performing and reading an mRDT, management of a patient with fever and either a positive or negative mRDT as well as patient communication. All health workers were invited to attend the training	Supervision at 6 weeks and 6 months	mRDTs supplied by MoH, with study back up in the case of stockouts	Training on patient-centred services; training in-charges in health centre management
Uga2/a	Four day interactive training, covering performing and reading an mRDT, how to prescribe antimalarials, how to deal with negative cases and communication skills. Providers were also given pictorial job aids	Close supervision for first 6 months (prior to evaluation)	mRDTs and ACTs supplied by study	Community sensitisation
Uga2/b				
Uga3	Four day interactive training to all drug shop vendors, which covered performing and reading mRDTs, prescribing antimalarials, how to deal with mRDT negatives and communicating and negotiating with patients	Close supervision for first 2 months (prior to evaluation)	mRDTs and ACTs supplied by study	Community sensitisation

Three studies compared different training packages^Nig1,Cam1,Tanz2^. Six studies compared intervention effects in different epidemiological contexts^Uga2,Tanz1,Nig1,Cam1,Afgh1,Ghan1^. Seven studies evaluated an intervention against a control arm where mRDTs were not made available^Uga1,Uga2,Uga3,Nig1,Cam1,Afgh1, Ghan1^.

### Comparability of findings

Although the studies were co-designed and largely similar, because of differences in primary study questions and differences in epidemiology, data collection methods and evaluation timing, mean pooled analyses would be inappropriate. For example, mRDT uptake was reported through provider-completed registers in some projects and patient exit interviews in others. Some studies reported adherence in terms of the percentage of patients *prescribed* ACTs or antimalarials, while others reported the percentage of patients who *received* them. Stockouts may have affected receipt of medication; whether prescriptions were affected is unknown, as alternative medication may or may not have been offered when there was a known stockout. The analysis presented therefore focuses on understanding the reasons for variation in the results, rather than seeking pooled point estimates.

Quantitative outcome data were extracted from each study's raw data set and reanalysed to maximise comparability across studies, using the most comparable denominators and numerators possible. Study, intervention and context characteristics were extracted from published and unpublished documents. Where available, thematic content analysis was undertaken on qualitative data from providers involved in the studies (ie, focus group discussions^Uga2,Uga3^ or interviews^Afgh1,Ghan1,Tanz1/a,Tanz1/b,Tanz2,Uga1^ with health workers, drug shop vendors or volunteers). In Tanz3, interviews from a later, related study were analysed, which included six study providers and six similar providers who had not been involved in the study but had comparable mRDT experiences.

The analysis drew on the approaches informing intervention component analysis (ICA)^52^ and qualitative comparative analysis (QCA),^53^ which seek to identify critical features of interventions. As with ICA, we sought to identify how interventions differed from one another and then, as with QCA, identify which factors appeared to be important. Our initial stage involved gathering as much information about the interventions as possible, going broader than the ICA approach by also capturing information about their delivery and context. However, our analysis differed from ICA and QCA, which attempt to characterise and apply scores to interventions and their characteristics and cross-tabulate these with outcomes. We found our data were not amenable to scoring in a quantitative sense, due to wide variation in the extent and types of information available. Therefore, our analysis was qualitative, using a meaning-based approach. Tables were created for each outcome of interest, with explanatory factors relating to the intervention, context and study design (see online supplementary file 2 for an example). These were shared with study teams and the ACT Consortium core scientific team, with ongoing discussions about the findings and other potential explanatory factors.

10.1136/bmjopen-2016-012973.supp2 supplementary file

## Results

**Figure 1:**
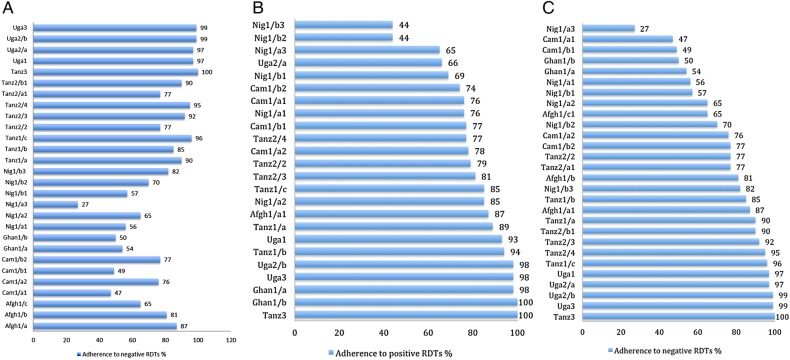
(A) Uptake of malaria rapid diagnostic tests (mRDTs) (% patients with fever or history of fever who were tested for malaria with an mRDT).(B) Adherence to positive mRDT results (% of patients with a positive mRDT who did receive ACTs). (C) Adherence to negative mRDTs (% of patients with a negative mRDT results who did NOT receive antimalarials).

There was wide variation across cases in all three outcomes: 12–100% mRDT uptake (figure 1A); 44–98% adherence to positive mRDTs (figure 1B); 27–100% adherence to negative mRDTs (figure 1C). All outcomes were universally high in some cases^Uga1,Uga2/b,Uga3^ and universally low in others^Nig1/a1,Nig1/a3^, but in many cases, the three outcomes did not correspond—for example, testing was infrequent but adherence to results high^Tanz1/a,Tanz1/b,Tanz2/3^ or adherence to positives high, but negatives low^Ghan1/a,Ghan1/b,Cam1/a1,Cam1/b1^, or vice versa^Uga2/a,Nig1/b3^.

There were no single factors which alone accounted for any of the outcomes; successful mRDT uptake and adherence appeared to result from a combination of context and intervention characteristics. The analysis identified several factors which, taken together, may account for the heterogeneity observed. The appeal of the intervention to providers was crucial for all three outcomes, but each was additionally shaped by other factors.

### Factors affecting mRDT uptake

There was wide variation between cases in the use of mRDTs for febrile patients (see figure 1A). Providers' motivation to perform well in the intervention was associated with uptake, as were familiarity with testing, adequate human resources and supplies, and the cost of mRDTs.

#### Motivation to perform well in the intervention

The range of sectors and contexts in which providers worked meant that their own priorities varied between cases. For example, government health workers' priorities may have included some or all of the following: treating ill patients, managing their workload in the light of staff shortages, managing (or ‘rationing’) their medicine supplies in the face of future shortages, maintaining their position of authority as a clinician. In contrast, while private providers may also have prioritised treating ill patients, some viewed their role as more of a business than a healthcare service. As such, their priorities may have been more business-oriented, such as making a profit and ensuring sufficient customers.

Data on provider priorities were not available for all cases; for some, qualitative data were available but for others, anecdotal evidence and study team perceptions were used. Nevertheless, where the intended use of mRDTs and associated intervention activities aligned well with providers' own priorities, they appeared more motivated to participate and ‘perform’ well in the intervention, and we observed higher uptake and adherence. There were a number of explanations for, and/or factors associated with, higher motivation but political and financial support were often critical. For example, in Tanz2, carefully developed messages addressing existing provider principles and practices, as well as Ministry of Health branding of the intervention (an institution known to influence the government health workers in this setting), appeared to motivate providers. In Uga3, the drug shop vendors were previously not permitted to offer testing and this new service, along with the associated training, supervision and visible involvement of the Ministry of Health, gave them a legitimacy they had previously lacked.^48^ These vendors also reported increased customer numbers and associated profits, enhanced by the study's free provision of mRDTs and ACTs for them to sell at a subsidised rate. In Tanz3, government providers were paid a supplement to participate in the study. Additional unintentional aspects of studies, such as regular visits or perceived support from evaluators, may have also helped to improve outcomes^Uga3,Tanz2^.^38^

In contrast, where mRDT interventions were not aligned with provider priorities, we saw lower uptake and adherence. For example, in Nig1 in the private sector, providers saw themselves more as vendors than healthcare practitioners. Here, there were anecdotal reports that they were particularly concerned about losing money from sales if mRDT results were negative and wondered whether the public would consider them legitimate to test. This was the case in spite of the free provision of mRDTs to providers by the study team. When providers viewed the intervention as extra unpaid work (eg, conducting tests or recording test results), this affected their motivation. In Uga3, some drug shops declined to participate in the trial for this reason and in Uga1, some health facilities hesitated to continue participating when they felt the work was too much without remuneration. Here, a misalignment between the providers' priorities and the intentions of the intervention led to a lack of motivation for providers to perform in line with guidelines.

#### Familiarity with testing

In most cases, there was little prior experience of malaria testing, either using mRDT or microscopy. Although patients were generally keen to be tested for malaria, it was not typically part of providers' routine habits to test. In cases where testing had become part of the established process of care, mRDT uptake tended to be higher. For example, in Tanz1/c, mRDTs had already been scaled up in other districts in recent years, and at baseline there was substantial microscopy testing, unlike the other two cases in this study where uptake was lower^Tanz1/a,Tanz1/b^. Wide-scale public awareness of testing may have facilitated uptake, for example, in Cameroon, where mass communication campaigns coincided with the study^Cam1^, which saw an increase in malaria testing in all study arms from baseline.^23^ Some interventions incorporated local community sensitisation activities to increase familiarity^Uga2,Uga3,Tanz2/4,Nig1/3^, although this appeared insufficient on its own to ensure high uptake.

#### Adequate human resources and supplies

Where staff workload was high, or patient numbers exceeded capacity, particularly in small facilities with only one staff member, mRDTs were not always used^Uga1,Tanz2/1^.

There were adequate stocks of mRDTs in facilities in most studies, in several cases due to study provision of additional supplies to avert stockouts. However, stockouts did occur in some studies^Cam1,Tanz1,Tanz2^, which was associated with lower uptake to some extent. Nevertheless, even when mRDTs were available, they were not always used, suggesting other factors were also influential.

#### Cost of mRDTs to patients

In most studies, mRDTs were provided free to patients. In those cases where providers were permitted to charge patients for mRDTs, higher prices may have affected their uptake. For example in Nig1, where mRDT uptake was among the lowest observed, patients were charged more than the recommended price on average, particularly in the private sector.

### Factors affecting adherence to positive mRDT results

ACTs were not consistently prescribed to patients with positive mRDT results (see figure 1B). Given the expectation for antimalarial overuse based on previous data, this finding was not anticipated and reasons for low adherence to positive results were therefore not explicitly explored during the studies. However, some explanatory factors driving this outcome did emerge, in addition to the motivation to perform well in the intervention (discussed above). These were the stability of ACT supplies and local preferences for different types of antimalarial.

#### Stability of ACT supplies

Stockouts of ACTs were associated with variation in adherence to positive mRDT results; however, this could not explain all the variation. In some cases, ACT use was relatively low despite no or few stockouts, whereas in others, use was high despite stockouts occurring. It may be that provider confidence in the stability of ACT supplies also influenced the use and rationing of ACTs, even when ACTs were available. For example, in Tanz2, lower rates of adherence to positive mRDTs were observed in the case where stockouts were most frequent^Tanz2/4^, even after periods of stockouts were excluded from the analysis.

#### Pre-existing antimalarial preferences

Information on pre-existing antimalarial preferences was gathered from baseline and preintervention surveys,^32^ ^49^ interview transcripts^Tanz1^ and unpublished reports,^54^ although no data were available for five studies^Afgh1,Ghan1,Tanz3,Uga1,Uga2^. The data suggest an association between the use of ACTs for positive mRDTs and baseline preferences for, or use of, ACTs rather than other antimalarials. For example, in Nig1, where ACT use was generally low, prior to the intervention, other antimalarials were asked for by patients, prescribed and purchased more commonly than ACTs.^34^ In contrast, in Tanz1, where adherence to RDT positive results was higher, according to stakeholder interviews, ACTs were patients' preferred antimalarial. This may have been due to greater exposure to community sensitisation around ACTs^55^ or cultural norms around provider authority such that patients felt more inclined to change their preferences in the light of providers' guidance than was the case in Nigeria. An alternative explanation relates to the different roles of the public sector in these countries and therefore, the different influence that the choice of official first-line medicines has on preferences. For example, in Tanzania, public facilities are much more widely used that they are in Nigeria, so people will have become used to the idea of ACTs. In Nigeria, the public sector is a more limited provider, so making a drug officially first line may have much less effect on preferences.

### Factors affecting adherence to negative mRDT results

There was also wide variation in the proportion of patients prescribed or given antimalarials in spite of negative mRDT results (see figure 1C). In addition to being motivated to perform well in the intervention (discussed above), the analysis suggests adherence to negative mRDTs was also driven in part by the extent to which mRDTs fitted—or were helped by intervention activities to fit—into the existing landscape of care (existing diagnostic and consultation practices). This included providers' perceptions of the role of mRDTs in the diagnostic process and possibilities for alternative diagnoses and treatment. In addition, the analysis suggests that adherence was affected by the extent to which the interventions attempted to control clinical practice.

Malaria tests were usually the only diagnostics available in study facilities. In most cases, test-based malaria diagnosis required a substantial shift from reliance on clinical judgement. In a minority of cases, this shift had already begun before the evaluation started, for example, in Tanzania and Zanzibar where mRDT introductions had begun nationally^Tanz1,Tanz3^, or where malaria testing using microscopy was established^Afgh1/a,Afgh1/b,Tanz1/c^. Here, mRDTs appeared to fit into the landscape of care more easily and adherence to negative mRDT results was higher. Where testing was new and did not fit into the landscape of care so well, even if mRDT use was attractive, adhering to negative results appeared more difficult^Afgh1/c,Cam1,Ghan1,Nig1^.

Two factors appeared to facilitate integration of mRDTs into the landscape of care: providers' perceptions of the role of mRDTs in the diagnostic process and whether alternative management of illnesses, not involving antimalarials, was possible for those with negative mRDT diagnoses.

#### Perceived role of mRDTs in diagnostic process

Two main factors influenced providers' perceptions of the role of mRDTs within the process of malaria diagnosis: how well mRDTs fitted with the dynamic of consultations and whether the mRDT results matched their expectations.

In some cases, providers saw mRDTs as central to the diagnostic process. For example, community health volunteers in Uga2, whose adherence was very high, described the mRDTs as working as ‘a judge’, and drug shop vendors in Uga3 saw taking blood as crucial to their enhanced role. Conversely, some providers felt clinical judgement should play a more important role in making a diagnosis than mRDTs. Qualitative data suggested that where mRDTs challenged clinicians' expertise and disrupted traditional consultation practices, this led to lower adherence to negative results ^Afgh1,Ghan1,Tanz2/1^. By questioning the test's accuracy, providers were able to reassert their authority and manage the consultation as usual.^18^ ^36^

Some interventions aimed to help mRDTs ‘fit’ with the dynamics of consultations. For example, training included role-play activities or reflections about how mRDTs would work in practice^Cam1/2,Uga1,Uga3^, experimentation^Tanz2/3. Tanz2/4^ and reflection facilitated by multiple training and feedback sessions with peers^Cam1/2,Tanz2/3,Tanz2/4,Uga1,Uga2,Uga3^; and training on communicating with patients^Cam1/2,Nig1/2, Tanz2/3,Tanz2/4,Uga1,Uga 2,Uga3^. Providers reported positive impressions of the training's impact on their interactions with patients including the importance of talking to patients and explaining the need for mRDTs or the meaning of their results^Ghan1,Tanz2/1,Tanz2/3,Tanz1/a,Uga2^.

In some cases, mRDT results did not match expectations; typically, fewer mRDTs were positive than had been expected, particularly when the tests were first introduced^Uga3,Tanz2/4,Ghan,1/2^. When this happened, providers placed less emphasis on mRDTs in the diagnostic process, preferring to rely more heavily on clinical judgement. For example, in Cam1/a1, mRDT positivity rates were just 9%, despite the local perception that malaria prevalence was high in that area. Several interviewees from different cases explained that it was hard to trust mRDTs when so many results were negative^Ghan1/b,Nig1,Tanz1/b,Tanz2/4,Uga3^, or that they only trusted them once they had seen some positive mRDT results^Uga2,Tanz2/4^. Providers described a fear of missing malaria diagnoses, particularly when the frequency of positive results was lower than expected, and this was associated with lower adherence^Ghan1/1,Ghan1/2,Tanz1/b^. In contrast, providers in Tanz3, where adherence to negative mRDTs was high, appeared less concerned about malaria, recognising that prevalence had declined. Some interventions explicitly aimed to raise awareness of current malaria epidemiology during training^Tanz2/3,Tanz2/4,Uga1^ in order to (re)set expectations of mRDT positivity rates; this was also associated with higher adherence to negative results.

In several cases, providers reported that their trust in mRDTs grew over time^Tanz3, Tanz2/2, Tanz2/3, Uga3^. Some described deliberate ‘experimentation’ to build trust in results, either by testing with microscopy as well as mRDTs^Afgh1^ or by seeing whether mRDT-negative patients recovered without antimalarials^Ghan1,Uga2^. Indeed in one study, this was explicitly encouraged^Tanz2/3, Tanz2/4^. Conversely, some providers' accounts showed mistrust of mRDTs was reinforced by experiences of seeing patients, or indeed themselves, recover when taking antimalarials in spite of a negative mRDT result^Uga2/b,Ghan1/a^. Patient follow-up was considered another useful means of building trust^Uga2, Ghan1/b^. Two interventions aimed to increase the perceived role of mRDTs by providing information about mRDTs' sensitivity and specificity^Tanz1,Tanz2/3,Tanz2/4^.^36^

#### Alternative treatments for non-malarial fever patients

Interventions offered different options for dealing with mRDT-negative patients (as mentioned above, data on the use of alternative treatments are presented in a separate paper). It appeared that expectations and options for alternative management of negative cases—in terms of providers' role, knowledge of case management and availability of other medicines—were important in antimalarial prescribing to mRDT-negative patients. In the public facility interventions where detailed guidance was given to aid alternative diagnoses^Uga1,Tanz2,Tanz3^, adherence was higher than in public facilities where no substantial guidance was provided^Ghan1,Afgh1^ or where it was recommended that providers only offer antipyretics to mRDT-negative patients^Nig1/2,Nig1/3^. At the community level, where volunteer providers were not expected (or permitted) to provide medicines beyond antimalarials^Uga2^, adherence to negative results was high. In private shops in Uganda, where no training on non-malarial febrile illness management was provided, adherence to mRDT-negative results was still high in terms of ACT prescription, although here mRDT-negative patients ended up being sold other medicines^Uga3^.

#### Directive intervention approach

Some interventions were more directive about provider practices, particularly regarding the use of unambiguous guidance and supervision or surveillance.

Adherence was typically higher if interventions instructed that no antimalarial should be given to those with negative mRDT results^Uga1,Uga2,Uga3,Tanz3^. In contrast, adherence was lower when an intervention allowed exceptions for when antimalarials could be given in spite of a negative result, for example, if a febrile patient was under 5 years and had travelled a long distance to seek care^Afgh1,Tanz2/2,Cam1^.

The highest adherence was observed among providers who had been closely supervised—either for an intense period after training^Uga2,Uga3^ or throughout the evaluation period^Tanz3^. Providers receiving feedback by text message experienced these as a form of surveillance, and reported responding by feeling they should follow guidelines even if their clinical judgement was at odds with this^Tanz2/3,Tanz2/4^.

## Discussion

This analysis addresses the persisting gap in knowledge around how to change prescribing practices. This is a key question in this time of international concern over resistance to antimicrobial medicines, with the imperative to optimise medicine use agreed on by United Nations signatories.^56^ ^57^ By analysing indepth data from 10 co-designed intervention studies from the ACT Consortium, we identify factors affecting the uptake of mRDTs and adherence to test results in different contexts. The varied findings suggest that to improve prescribing through mRDTs, interventions must go beyond basic training in mRDT use and must be tailored to the needs of providers in particular contexts. Uptake and adherence were highest where providers were motivated by the intervention and the tests fitted with the landscape of care. Intervention characteristics that aligned mRDTs with provider priorities included interactive training that addressed how to manage test-negative patients in practice, including clinical and interpersonal aspects of care. Where malaria endemicity is overestimated locally, experimentation and feedback on frequent test-negative cases was important. A directive approach supported by feedback or supervisory instruction can yield high adherence to guidelines but may affect patient-centred care. The results suggest that as mRDTs become established, the intensity of supporting interventions required is likely to reduce.

A strength of this analysis was its use of rich data sources which enabled a more indepth and comprehensive analysis. Although additional insights may have emerged from inclusion of a wider set of studies, synthesising findings from published healthcare interventions is often challenging, with diverse and poorly described interventions, contexts and methods.^58^ ^59^ Nevertheless, our analysis was limited by the fact that not all included studies were able to provide information on all characteristics of interest, while for other characteristics (eg, year and duration), there was too much variation to identify any patterns. While study samples were generally sizeable, in some cases where testing rates and/or malaria prevalence were low, the denominator for adherence outcomes was small. With one exception, where a government mRDT policy was evaluated^Tanz1^, all of the evaluated interventions in this analysis were instigated by the study teams. As such, there may be aspects of the interventions, such as RDT supply sources and costs to providers, which may not apply at scale.

Previous studies have identified capacity issues as important in mRDT implementation, such as staffing levels or overworked staff,^9^ ^12^ ^60–64^ mRDT or ACT supplies,^9^ ^12^ ^61–65^ and providers' confidence in mRDT results.^12^ ^61–66^ Our synthesis shows that beyond these issues, the introduction of the tests had to *make sense* in context. Some interventions in our analysis additionally included a more directive approach. While these interventions did achieve the highest rates of adherence to negative results, the consequences of restricting the autonomy of clinicians in favour of standardised guidelines need to be weighed up against the need for clinicians to consider individual patients on a case-by-case basis.^67^ Our finding, that settings where testing was more familiar used mRDTs more appropriately, echoes observations from country-level roll-out of mRDTs,^68^ ^69^ and suggests that the interventions required will change over time. Our finding, that basic training alone is insufficient to ensure use of the tests as intended, aligns with findings from studies of interventions aiming to change clinical practice in general.^4^ ^70^

Prior to introducing mRDTs, initial assessments should be carried out to understand providers' priorities and capacities, as well as how easily tests might integrate into landscapes of care. Although our analysis suggests that a process of tailoring is required to formulate the intervention to best fit each context, certain broad intervention features are likely to be applicable across settings (see box 1). As these recommendations arise directly from the data available in our studies, they are not exhaustive.

Box 1 **Examples of recommended intervention features**

Planning

Recognise and address providers’ priorities

Staffing

Ensure sufficient staff numbers for increased workload

Training

- Offer longer, more detailed training, incorporating interactive activities
- Include training on communicating with patients
- Address process of change to test-based care:
  plan a series of interactive training and/or supervision sessions
  incorporate role-play activities which address local challenges
  use reflective activities
- Build trust in mRDTs by including:
  discussion of data on changes in malaria prevalence in the area
  discussion of sensitivity and specificity of mRDTs
  encouragement to cross-check these data with experience of tests in practice

Guidance

- Provide detailed guidance and resources for acceptable case management for mRDT-negative patients
- Consider how directive mRDT guidance should be, balancing clarity with the need for clinician judgement to make exceptions (eg, if patients have travelled far, with limited means of transportation to return if their condition worsens)

Medical supplies

- Ensure providers can be confident in supplies of mRDTs and ACTs
- Keep costs to patients low

Community/patient sensitisation

- Conduct patient-oriented sensitisation activities
  where familiarity with testing is low, where frequent false-positive microscopy has overestimated prevalence, or if ACTs are not the most common antimalarial used or demanded by patients

These findings can inform broader antimicrobial stewardship efforts. Malaria is the first disease for which interventions have been systematically evaluated in order to understand how to change routine prescribing through rapid diagnostics. The lessons learnt in attempting to shift from presumptive to test-directed treatment are relevant for interventions beyond malaria. The intervention and contextual characteristics identified here highlight that apparently simple technological solutions can require complex supporting apparatus when implemented in real life.^71^ However, these findings suggest that as mRDTs become established, the intensity of supporting interventions required is likely to reduce. Further research could explore whether an initial investment in mRDTs could establish patterns of care that allow for other diagnostic tests to be introduced more easily in the future.

### Conclusion

This analysis shows that uptake and adherence to mRDTs can be high, but this requires either existing contexts where integrating the tests into practice already makes sense, or tailored interventions to encourage this. Basic training and supplies are essential but insufficient to maximise the potential of mRDTs in contexts where they do not fit well with the landscape of care. Apparently simple technological solutions such as mRDTs can require complex supporting interventions that take account of how they will be interpreted and used.
