# Supplementary material for: Improving prescribing practices with rapid diagnostic tests (RDTs): synthesis of 10 studies to explore reasons for variation in malaria RDT uptake and adherence
Source: BMJ Open. 2017 Mar 8;7(3):e012973. doi: 10.1136/bmjopen-2016-012973 (PMC5353269; doi:10.1136/bmjopen-2016-012973)
Supplement: supplementary file [file bmjopen-2016-012973supp1.pdf]

Supplementary file 1: Descriptions of studies included

Table of Contents

Afgh1: Strategies for expanding access to quality malaria diagnosis in south-central Asia where malaria incidence is low 2

Cam1: Cost-effectiveness of interventions to support the introduction of malaria rapid diagnostic tests in Cameroon 6

Ghan1: How the use of rapid diagnostic tests influences clinicians' decision to prescribe ACTs 10

Nig1: Costs and effects of strategies to improve malaria diagnosis and treatment in Nigeria 14

Tanz1: IMPACT 2: Evaluating policies in Tanzania to improve malaria diagnosis and treatment 19

Tanz2: Targeting ACT drugs: the TACT trial 23

Tanz3: Trusting rapid diagnostic tests in Zanzibar 28

Uga1: The PRIME trial: Improving health centres to reduce childhood malaria in Uganda 31

Uga2: Use of rapid diagnostic tests to improve malaria treatment in the community in Uganda 35

Uga3: Introducing rapid diagnostic tests in drug shops to improve the targeting of malaria treatment 39

Afgh1: Strategies for expanding access to quality malaria diagnosis in south-central Asia where malaria incidence is low

Location	Afghanistan
Sector targeted	Public
Intervention dates	September 2009 – September 2010
Timing of evaluation	September 2009 – September 2010
Prescriber sample	Afgh1/a: 12 clinics<br>Afgh1/b: 5 clinics<br>Afgh1/c: 5 clinics
Patient sample	Afgh1/a: 1,576<br>Afgh1/b: 516<br>Afgh1/c: 324
Qualitative data collected from prescribers?	Yes

<u>Background context</u>

The study took place in secure, rural areas. There were very high rates of malaria over-diagnosis at baseline in all scenarios, particularly in Afgh1/c. The most common form of malaria was *P. vivax* rather than *P. falciparum*. The prevalence of malaria was perceived to be high in all scenarios. mRDTs had not been scaled up prior to the intervention.

Cases:
Afgh1/a: Eastern province, established microscopy
Moderate malaria transmission rates. All 12 study clinics had microscopy installed for more than 5 years. Clinics in this region were generally busier than those in the northern region, seeing more patients.

Afgh1/b: Northern province, new microscopy
Low malaria transmission rates. All five study clinics had recently established microscopy (since 2009). Clinics in this region were generally quieter than those in the eastern region, seeing fewer patients.

Afgh1/c: Northern province, no microscopy
Low malaria transmission rates. All five study clinics had no microscopy and relied on clinical diagnosis prior to the intervention. Clinics in this region were generally quieter than those in the eastern region, seeing fewer patients.

Intervention

Patients were randomised to receive either an mRDT or usual care (either microscopy, in Afgh1/a and Afgh1/b, or clinical diagnosis in Afgh1/c).

mRDTs were supplied by the project and were free to clinics and patients. 1.5 days training was offered to all facility staff, following the national training package. This covered performing mRDTs (most, but not all, practiced testing) and prescribing antimalarials, but not how to treat patients with negative mRDT results.

**Medical supply mechanism**	mRDTs supplied by study; ACTs through standard mechanism
**Were continuous supplies assured?**	Yes, for RCTs. Not for ACTs.
**Cost of mRDTs/ACTs to patients**	Free
**Who conducted mRDT?**	Prescriber

Study design

Two-arm patient randomised controlled trial. All clinics in the study areas participated in the intervention.

mRDT uptake was not assessed as patients enrolled in the study were randomised to receive an mRDT or standard care (microscopy or presumptive diagnosis, depending on the scenario). Patients were enrolled in the study if they gave informed consent and had a fever or a self-reported history of fever in last 48hrs, where the clinician suspected malaria and would normally request a diagnosis or treat with a malaria drug. Patients were excluded if the patient had a diagnostic result from another health facility, if the clinician provided treatment without testing or if, the clinician specifically requested a blood slide. Data was collected from project-specific registers completed by prescribers.

Findings

ACT received when positive mRDT:
Afgh1/a: 87%
Afgh1/b: n/a (no Pf identified)
Afgh1/c: n/a (no Pf identified)

Antimalarial received when negative mRDT:
Afgh1/a: 13%
Afgh1/b: 19%
Afgh1/c: 35%

Possible explanatory factors:

- **Familiarity with testing**: adherence to negative results was higher in both scenarios where microscopy was available. In interviews, health workers often did not appear to distinguish between mRDTs and microscopy.
- **Poor fit with landscape of care**: tests were felt to be good at confirming clinical diagnosis of malaria, but clinical diagnosis could overrule a negative test (which represented an 'absence of diagnosis').
  Health workers did not always trust mRDTs, possibly because they challenged clinicians' autonomy. The training did not attempt to convince health workers of the accuracy of mRDTs (e.g. by presenting results from local research into their accuracy). Some health workers who were interviewed explained that they didn't trust mRDTs because the tests were being studied in the trial i.e. they misunderstood the purpose of the trial and thought that the mRDTs themselves were being studied.
  There was low malaria prevalence (and so no/few positive mRDTs) which was not expected.
- **Low acceptability of alternatives to antimalarials**: There was a perception among health workers that they did not want to miss a malaria diagnosis and that antimalarials were fairly benign. The training did not cover how to deal with negative cases.
- **Intervention messages:** guidelines were perceived to be incongruent with mRDT adherence. Health workers interviewed reported that IMCI guidelines stated they should give antimalarials if a child was feverish or no there were no signs of other diseases, even if an mRDT was negative. Ministry of Health guidelines included three categories of diagnosis: confirmed malaria, suspected malaria and negative for malaria. 'Suspected malaria' was expected to be used in situations where no testing facilities were available, although health workers believed they could use this in other circumstances too, e.g. if typical signs and symptoms of malaria were displayed, with no other disease symptoms.
- **Mixed motivation to perform well in the intervention:** Some health workers who were interviewed explained that they didn't trust mRDTs because the tests were being studied in the trial (i.e. they misunderstood the purpose of the trial and thought that the mRDTs themselves were being studied) .

- **Study design**: in interviews, some health workers explained that they didn't send patients for testing (i.e. recruit them into the study) if they displayed typical signs & symptoms of malaria. One health worker from the northern province, new microscopy scenario mentioned in an interview that they didn't test if they had a heavy workload.

Related publications

1. Leslie T, Mikhail A, Mayan I, Anwar M, Bakhtash S, Nader M, et al. Overdiagnosis and mistreatment of malaria among febrile patients at primary healthcare level in Afghanistan: observational study. *BMJ*. 2012;345:e4389.
2. Reynolds J, Wood M, Mikhail A, Ahmad T, Karimullah K, Motahed M, et al. Malaria "diagnosis" and diagnostics in Afghanistan. *Qual Health Res*. 2013;23(5):579-91.
3. Leslie T, Mikhail A, Mayan I, Cundill B, Anwar M, Bakhtash SH, et al. Rapid diagnostic tests to improve treatment of malaria and other febrile illnesses: patient randomised effectiveness trial in primary care clinics in Afghanistan. *BMJ*. 2014;348:g3730.

Cam1: Cost-effectiveness of interventions to support the introduction of malaria rapid diagnostic tests in Cameroon

Location	Cameroon:<br>Bamenda (northwest, rural & urban)<br>& Yaoundé (central, urban)
Sector targeted	Government and mission
Intervention dates	June – December 2011
Timing of evaluation	September – December 2011
Prescriber sample	Cam1/1a: 8 facilities<br>Cam1/1b: 10 facilities<br>Cam1/2a: 9 facilities<br>Cam1/2b: 10 facilities
Patient sample	Cam1/1a: 403<br>Cam1/1b: 402<br>Cam1/2a: 552<br>Cam1/2b: 311
Qualitative data collected from prescribers?	No

Background context

Malaria was endemic in both settings. At baseline, microscopy was available in almost all health facilities but mRDTs were not. Clinical diagnosis was the common method of malaria diagnosis, with local clinical guidelines recommending presumptive treatment as default course of action. These also stated that fever was the most reliable symptom for treatment and diagnosis and that a negative microscopy result did not rule out malaria. Health workers did not consider testing to be very important for patients and did not themselves feel it was acceptable to withhold antimalarials if a patient tested negative. Overdiagnosis was common, with 81% of febrile patients receiving antimalarials although only 35% of these had malaria. mRDTs had been in the national guidelines since 2008 although there were reports that local clinical guidelines still recommended presumptive treatment.

Cases:

Cam1/1a: intervention 1, Bamenda
Cam1/1b: intervention 1, Yaoundé
Cam1/2a: intervention 2, Bamenda
Cam1/2b: intervention 2, Yaoundé

Intervention

Intervention arm 1
mRDTs provided with basic training
Four prescribers per cadre per facility were invited to attend a one day training; they were strongly encouraged to train others in their facilities. This didactic session covered three modules: malaria diagnosis, mRDTs, and malaria treatment. The study team conducted monthly supervisory visits and provided 100 mRDTs to each facility per month, which were sold to patients (at a higher rate than the US$0.20 per test the project had requested) or provided free to under 5s.

Intervention arm 2
mRDTs provided with basic and enhanced training
In addition to the interventions provided in arm 1, an interactive two day training was delivered that was designed to change prescribing practices. This covered adapting to change (focused on WHO malaria treatment guidelines), professionalism, and effective communication.

**Medical supply mechanism**	mRDTs & ACTs supplied by study
**Were continuous supplies assured?**	No: 100 mRDTs supplied for each facility per month
**Cost of mRDTs/ACTs to patients**	RRP US$0.20 (free to U5s)<br>Mean actual price of mRDT:<br>Cam1/1: US$1.28<br>Cam1/2: US$2.09
**Who conducted mRDT?**	Prescriber

Study design

Three-arm cluster randomised controlled trial. The control arm did not have mRDTs and was not included in the current analysis.

Facilities were eligible for inclusion in the study if they were not part of a government pilot roll-out of mRDTs, if they did not offer specialist services, if they received more than four febrile patients per day on average and if they were more than 2km away from another facility in Bamenda or more than 1km away in Yaoundé.

Evaluation started three months after intervention and ran for three months. Data was collected from project-specific registers completed by prescribers, as well as patient exit interviews. Fieldworkers collected registers from facilities

each week. All patients who attended the health facilities were approached on exit for consent to participate in the study and screened for eligibility. Patients were eligible for inclusion in the exit survey if they reported seeking treatment for fever or suspected malaria. Patients were excluded if they were pregnant, younger than six months, or had signs of severe malaria. Individuals were also excluded if the patient was not present.

Findings

Uptake (% of febrile patients, who were not tested with microscopy, who were tested with an mRDT)
Cam1/1a: 49%
Cam1/1b: 60%
Cam1/2a: 72%
Cam1/2b: 45%

Adherence to positive mRDT
(% of patients testing positive with an mRDT who received ACTs)
Cam1/1a: 76%
Cam1/1b: 77%
Cam1/2a: 78%
Cam1/2b: 74%

Adherence to negative mRDT
(% of patients testing negative with an mRDT who did NOT receive any antimalarials)
Cam1/1a: 47%
Cam1/1b: 49%
Cam1/2a: 76%
Cam1/2b: 77%

Possible explanatory factors:

- **Patient expectations of malaria testing** - a concurrent extensive malaria communication campaign (external to the intervention being evaluated) had run after the formative research but before the evaluation, targeting testing and ACT use. Testing was also high in the control scenarios (higher than at baseline).
- **Familiarity with testing** - testing overall (microscopy or mRDT) was generally high in all cases, but it was slightly lower in Cam1/1a than the other cases (71% vs 78-81%)

- **Alternative treatments for non-malarial fever patients** - prior to the intervention, malaria was felt to be a well known, common and serious disease. Although testing was acceptable to patients (as a placebo), as was a positive malaria diagnosis, negative results were not considered acceptable and health workers reported finding it hard to give non-malaria diagnoses and treatments prior to the intervention.

Related Publications

1. Chandler CIR, Mangham L, Njei AN, Achonduh O, Mbacham WF, Wiseman V. 'As a clinician, you are not managing lab results, you are managing the patient': How the enactment of malaria at health facilities in Cameroon compares with new WHO guidelines for the use of malaria tests. *Social Science and Medicine*. 2012;74(10):1528-35.
2. Mangham LJ, Cundill B, Achonduh OA, Ambebila JN, Lele AK, Metoh TN, et al. Malaria prevalence and treatment of febrile patients at health facilities and medicine retailers in Cameroon. *Tropical Medicine & International Health*. 2012;17(3):330 - 42.
3. Wiseman V, Mangham LJ, Cundill B, Achonduh OA, Nji AM, Njei AN, et al. A cost-effectiveness analysis of provider interventions to improve health worker practice in providing treatment for uncomplicated malaria in Cameroon: a study protocol for a randomized controlled trial. *Trials*. 2012;13(4).
4. Achonduh OA, Mbacham WF, Mangham-Jefferies L, Cundill B, Chandler C, Pamen-Ngako J, et al. Designing and implementing interventions to change clinicians' practice in the management of uncomplicated malaria: lessons from Cameroon. *Malaria Journal*. 2014;13:204.
5. Mangham-Jefferies L, Wiseman V, Achonduh OA, Drake TL, Cundill B, Onwujekwe O, et al. Economic evaluation of a cluster randomized trial of interventions to improve health workers' practice in diagnosing and treating uncomplicated malaria in Cameroon. *Value Health*. 2014;17(8):783-91.
6. Mbacham WF, Mangham-Jefferies L, Cundill B, Achonduh O, Chandler CIR, Ambebila JN, et al. Basic or enhanced clinical training to improve adherence to malaria treatment guidelines: a cluster-randomised trial in two areas of Cameroon. *The Lancet*. 2014;2:346 - 58.
7. Mangham-Jefferies, L., K. Hanson, W. Mbacham, O. Onwujekwe and V. Wiseman (2014). "What determines providers' stated preference for the treatment of uncomplicated malaria?" Soc Sci Med **104**: 98-106.

Ghan1: How the use of rapid diagnostic tests influences clinicians' decision to prescribe ACTs

Location	southern Ghana
Sector targeted	Public and private health facilities
Intervention dates	August 2007 – December 2008
Timing of evaluation	August 2007 – December 2008
Prescriber sample	Ghan1/a: 1 facility<br>Ghan1/b: 3 facilities
Patient sample	Ghan1/a: 1,896<br>Ghan1/b: 1,719
Qualitative data collected from prescribers?	Yes

<u>Background context</u>

The intervention took place in the rural Dangme West district. Ghana is a country with high transmission of malaria, although incidence has been falling. The study took place before the country had introduced a policy to use malaria tests. Most of the healthcare professionals in the study sites were nurses with 2-3yrs of basic training.

Cases:
Ghan1/a: Microscopy scenario
One large health facility with a high patient load and with microscopy available. It had 16 prescribing staff.

Ghan1/b: Clinical diagnosis setting
Three facilities: One private clinic and two smaller public health facilities, with lower patient loads and no medical doctors (only medical assistances and nurses). These had no access to parasitological testing for malaria – diagnosis was based on clinical symptoms. They had 13 prescribing staff in total.

<u>Intervention</u>

All healthcare professionals in participating centres received two days of training on:

- The sensitivity and specificity of mRDTs
- Alternative causes of febrile illness
- The Ghana national guidelines (which indicate presumptive treatment for U5s)

They were left free to make their own clinical decisions after the initial training. New staff were given a one-to-one introduction to mRDTs using the same training package.

Included patients were randomised to receive an mRDT or standard care (microscopy or clinical diagnosis); uptake was not assessed.

**Medical supply mechanism**	mRDTs supplied by study; ACTs through standard mechanisms
**Were continuous supplies assured?**	Yes for RCTs, not for ACTs
**Cost of mRDTs/ACTs to patients**	Free
**Who conducted mRDT?**	Study staff, not prescriber

Study design

Two-arm patient randomised trial. All participants visiting the health facilities were screened for enrolment into the study. The inclusion criteria were that the healthcare professional considered treating the patient for malaria and wanted to test for malaria or treat the patient with an antimalarial. Exclusion criteria were pregnancy, illness severe enough to warrant referral to hospital, insistence by the health professional on a particular test or a particular method of treatment, patient insistence on a particular test, refusal of consent by patient/guardian, not living in the district or nearby, or not intending to remain in the district for the next two months for follow up.
Data was collected from a prescriber-completed register.

Findings

Adherence to positive mRDTs
(% of patients testing positive with an mRDT who received ACTs):
Ghan1/a: 98%
Ghan1/b: 100%

Adherence to negative mRDTs
(% of patients testing negative with an mRDT who did NOT receive any antimalarials):
Ghan1/a: 54%
Ghan1/b: 51%

Possible explanatory factors:

- **Poor fit with landscape of care** - prescribers continued to have faith in their ability to diagnose clinically. Prescribers didn't seem to take on board that they shouldn't give antimalarials if the mRDT was negative; possibly because of training and incongruence with guidelines (see below under 'intervention messages')
  - health workers initially noted differences between mRDT results and clinical diagnosis/microscopy results – which led to mistrust of mRDT for many.

  With time, observation of improvements in negatives not prescribed antimalarials/no improvement in negatives prescribed antimalarials (but challenge when patients don't return for follow up). Experimentation with changing their practice (convinced some but not others). Some health workers believed that malaria could 'hide' from the tests, e.g. if in the early stages of illness, if the patient has sickle cell, or because the parasite 'hides' in the liver. Some in the presumptive scenario explained the storage or handling of the test could affect its accuracy.
  Communities of practice influenced health workers – gave confidence in mRDTs for some; for others, gave confidence in clinical diagnosis.
- **Intervention messages:** national guidelines state presumptive treatment of U5s (incongruent with aim of testing)
- **Alternatives treatments for non-malarial fever patients:** health workers described that a common perception was that "in this country everything is malaria"; malaria was considered high prevalence and prescribers feared missing a malaria diagnosis and a patient dying because of this.
  In some cases, prescribers felt they had no choice but to meet the patient's wishes. Health workers perceived community members held onto the idea that all fever is malaria and preferred a malaria diagnosis; sometimes mistrusting health workers who gave a different diagnosis
  Patients reported conceptualising mRDTs as a generic test that should result in a diagnosis. The testing process was opaque for patients.
  Health workers highlighted the importance of communication for patient satisfaction/acceptance of mRDT results. However focus group discussions with patients found limited efforts by health workers to engage patients in the testing process and strong hierarchies leading to a lack of communication.

Related publications

1. Ansah EK, Narh-Bana S, Epokor M, Akanpigbiam S, Quartey AA, Gyapong J, et al. Rapid testing for malaria in settings where microscopy is available and peripheral clinics where only presumptive treatment is available: a randomised controlled trial in Ghana. *BMJ*. 2010;340:c930.
2. Chandler CIR, Whitty CJM, Ansah EK. How can malaria rapid diagnostic tests achieve their potential? A qualitative study of a trial at health facilities in Ghana. *Malaria Journal*. 2010;9(1):95.
3. Ansah EK, Reynolds J, Akanpigbiam S, Whitty CJM, Chandler CIR. “Even if the test result is negative, they should be able to tell us what is wrong with us”: a qualitative study of patient expectations of rapid diagnostic tests for malaria. *Malaria Journal*. 2013;12(1):258.

Nig1: Costs and effects of strategies to improve malaria diagnosis and treatment in Nigeria

Location	Enugu State, Southeast Nigeria
Sector targeted	Government, private pharmacies and patent medicine dealers
Intervention dates	June – December 2011
Timing of evaluation	September – December 2011
Prescriber sample	Nig1/1a: 38 facilities<br>Nig1/1b: 6 facilities<br>Nig1/2a: 38 facilities<br>Nig1/2b: 10 facilities<br>Nig1/3a: 36 facilities<br>Nig1/3b: 9 facilities
Patient sample	Nig1/1a: 1,182<br>Nig1/1b: 197<br>Nig1/2a: 1,396<br>Nig1/2b: 325<br>Nig1/3a: 906<br>Nig1/3b: 183
Qualitative data collected from prescribers?	No

Background context

The intervention took place in two areas: Enugu (an urban area) and Udi (a rural area). In Udi, 53% of included facilities were public, compared to 9% in Enugu. Malaria was endemic. Patent medicine dealers were a major source of treatment for malaria. Few primary health care facilities offered malaria testing at baseline. Few prescribers knew about mRDTs and test results were not always believed to be accurate. Patient demand for mRDTs was perceived to be low. Formative research in 2009 found antimalarial prescription for febrile patients was high (79%), although the majority were not given ACTs (only 23%). It was common for patients to ask for a specific drug; asking for ACTs was associated with a greater likelihood of receiving them.

Cases:
Nig1/1a: control arm, Enugu
Nig1/1b: control arm, Udi
Nig1/2a: intervention 1, Enugu
Nig1/2b: intervention1, Udi
Nig1/3a: intervention 2, Enugu
Nig1/3b: intervention 2, Udi

<u>Intervention</u>

Control arm: mRDTs with basic instructions
mRDTs were supplied free to prescribers by the study (and free to patients in public facilities; private facilities were asked not to sell them for more than US$0.6). 1-2 prescribers per facility were invited to attend a half-day demonstration on how to use mRDTs, which included practising conducting one test. They also received a copy of the WHO job aid, which shows the steps in using an mRDT. Staff from 77% of included facilities attended the training.

Intervention arm 1: mRDT with enhanced health worker training
25-75 mRDTs were supplied free by the study each month; prescribers could request more if they ran out (these were free to patients in public facilities, private facilities were asked not to sell them for more than US$0.6). 1-2 prescribers per facility were invited to attend a two-day interactive, seminar-style training, covering how to test, appropriate treatment for positive and negative results and effective communication. Those attending were given job aides (e.g. treatment algorithm). In addition, there were monthly supervisory visits with feedback on performance, as well as telephone support.

Intervention arm 2: mRDT with enhanced health worker training and school-based activities
In addition to intervention 1, primary and secondary schools were invited to send two teachers each for a 2-day training, who would then train six school children as peer health educators. Various activities would be run in schools and the local community to raise awareness about mRDTs for malaria and that ACTs were the recommended treatment for malaria. There were monthly support visits.

**Medical supply mechanism**	mRDTs supplied by study; ACTs were supplied through standard mechanism.
**Were continuous supplies assured?**	mRDTs – yes<br>ACTs – no
**Recommended cost of mRDTs to patients**	Public sector: free<br>Private sector: RRP US$0.60<br>Mean patient-reported price:
**Reported cost of mRDTs to patients: Nig1/1**	Public facilities: US$0.00 (0.00 – 2.1)<br>Pharmacy: US$0.60 (0.60-4.80)<br>Drug store: US$0.90 (0.30 – 7.20)
**Reported cost of mRDTs to patients: Nig1/2**	Public facilities: US$0.00 (0.00 – 1.2)<br>Pharmacy: US$0.60 (0.60-0.90)<br>Drug store: US$0.90 (0.30 – 3.00)
**Reported cost of mRDTs to patients: Nig1/3**	Public facilities: US$0.00 (0.00 – 0.30)<br>Pharmacy: US$0.90 (0.60-5.70)<br>Drug store: US$1.20 (0.30 – 7.20)
**Cost of ACTs to patients**	not subsidised; price unknown
**Who conducted mRDT?**	Prescriber

<u>Study design</u>

Three-arm cluster randomized controlled trial.

Clusters were defined as a geographical community containing at least one facility and one school. Schools and facilities were randomly selected within each cluster to receive the intervention. Up to three schools per cluster were selected. Private and government facilities were selected using probability proportional to size.

Data was collected from project-specific registers, completed by prescribers, as well as exit interviews with all eligible patients, which started three months after the intervention. Patients were eligible if they presented at the facility and they (or their caregiver) reported seeking treatment for fever or suspected malaria. Patients were excluded if they were pregnant, less than 6 months old, or had signs and symptoms of severe malaria.

Findings

Uptake
(% of febrile patients not tested with microscopy who were tested with an mRDT)

Nig1/1a: 25%
Nig1/1b: 46%
Nig1/2a: 20%
Nig1/2b: 37%
Nig1/3a: 12%
Nig1/3b: 51%

Adherence to positive mRDT
(% of patients testing positive with an mRDT who received ACTs)

Nig1/1a: 76%
Nig1/1b: 69%
Nig1/2a: 85%
Nig1/2b: 44%
Nig1/3a: 65%
Nig1/3b: 44%

Adherence to negative mRDTs
(% of patients testing negative with an mRDT who did NOT receive any antimalarials)

Nig1/1a: 56%
Nig1/1b: 57%
Nig1/2a: 65%
Nig1/2b: 70%
Nig1/3a: 27%
Nig1/3b: 82%

Possible explanatory factors:

- **Low motivation to perform well in the intervention** - anecdotal evidence suggested mRDTs were not accepted by prescribers for a range of reasons, such as concern in the private sector that consumers would not consider them legitimate to conduct the tests. mRDTs were viewed as having a negative impact on profits. A higher proportion of facilities in Enugu were private compared to Udi. Private sector prescribers charged more than the recommended retail price for mRDTs, which may have led to less demand for testing in private facilities.

- **Implementation -** less than half the schools in Nig1/3 organised a malaria event, which may explain the lack of difference in effect between Nig1/2 and Nig1/3
- **Poor fit with landscape of care -** anecdotal evidence that prescribers were surprised how many tests were negative and were not convinced of their quality or accuracy

Related publications

1. Mangham LJ, Cundill B, Ezeoke O, Nwala E, Uzochukwu BSC, Wiseman V, et al. Treatment of uncomplicated malaria at public health facilities and medicine retailers in south-eastern Nigeria. *Malaria Journal*. 2011;10:155.
2. Ezeoke OP, Ezumah NN, Chandler CC, Mangham-Jefferies LJ, Onwujekwe OE, Wiseman V, et al. Exploring health providers' and community perceptions and experiences with malaria tests in South-East Nigeria: a critical step towards appropriate treatment. *Malaria Journal*. 2012;11:368.
3. Wiseman V, Ogochukwu E, Emmanuel N, Lindsay JM, Bonnie C, Jane E, et al. A cost-effectiveness analysis of provider and community interventions to improve the treatment of uncomplicated malaria in Nigeria: study protocol for a randomized controlled trial. *Trials*. 2012;13:81.
4. Mangham-Jefferies L, Hanson K, Mbacham W, Onwujekwe O, Wiseman V. Mind the gap: knowledge and practice of providers treating uncomplicated malaria at public and mission health facilities, pharmacies and drug stores in Cameroon and Nigeria. *Health Policy & Planning*. 2014.
5. Mangham-Jefferies L, Hansen K, Mbacham W, Onwujekwe O, Wiseman V. What determines providers' stated preference for the treatment of uncomplicated malaria? *Social Science and Medicine*. 2014;104:98 - 106.

Tanz1: IMPACT 2: Evaluating policies in Tanzania to improve malaria diagnosis and treatment

Location	Tanzania<br>Tanz1/a: Mwanza<br>Tanz1/b: Mbeya<br>Tanz1/c: Mtwara
Sector targeted	Government primary care facilities
Intervention dates	Tanz1/a: February 2011 – (no end date)<br>Tanz1/b: February 2011 – (no end date)<br>Tanz1/c: May 2012 – (no end date)
Timing of evaluation	Tanz1/a: April/May 2012<br>Tanz1/b: May/June 2012<br>Tanz1/c: June/July 2012
Prescriber sample	60 health facilities in each case
Patient sample	Tanz1/a: 661<br>Tanz1/b: 347<br>Tanz1/c: 519
Qualitative data collected from prescribers?	Tanz1/a: Yes<br>Tanz1/b: Yes<br>Tanz1/c: No

Background context

Predominantly rural setting. The availability of microscopy and baseline malaria testing levels were higher in Tanz1/c compared to Tanz1/a and Tanz1/b. In addition, awareness of mRDTs was greater at baseline in Tanz1/c, possibly because national roll out of mRDTs had occurred in other parts of Tanzania before reaching there, providing time for awareness to be raised.

Cases:
Tanz1/a: Mwanza region: moderate-high malaria prevalence
Tanz1/b: Mbeya region: low malaria prevalence
Tanz1/c: Mtwara region: moderate-high malaria prevalence

Intervention

Phased national government roll out of mRDTs from 2009 – 2012. Training was the standard, two-day Ministry of Health (MoH) training, covering performing mRDTs (including practical) and prescribing antimalarials. One-two

staff per facility were invited to training and expected to pass information to colleagues.
Following supply of an initial stock of mRDTs to facilities, subsequent supplies could be ordered through standard MoH procedures.

**Medical supply mechanism**	mRDTs & ACTs supplied by government. Initial stock supplied, subsequent supplies ordered through standard MoH procedures.
**Were continuous supplies assured?**	No
**Cost of mRDTs/ACTs to patients**	Flat rate consultation fee, although some charged extra for diagnostics. There were exceptions, in theory, for some e.g. U5s.
**Who conducted mRDT?**	Prescriber

Study design

Observational study, with baseline data collection prior to introduction and one round of data collection at endline. Data was collected through patient exit interviews conducted on one day during daytime operating hours. Patient-held medical records were consulted if patients did not know what testing or treatment they had obtained. All patients with fever or history of fever in the past 48 hours who presented for outpatient care were enrolled in the study, subject to informed consent having been obtained.

All facilities were included in the intervention, as it was a national government roll-out. For this study, facilities were randomly selected for evaluation with probability proportional to malaria outpatient utilization for endline data collection. All patients with fever or history of fever in the past 48 hours who presented for outpatient care were included, subject to informed consent having been obtained.

Findings

Uptake
(% of patients with history of fever in past 48 hours who had not been tested with microscopy or an unknown test, who were tested with an mRDT)
Tanz1/a: 41%
Tanz1/b: 36%
Tanz1/c: 69%

Adherence to positive mRDTs
(% of patients testing positive with an mRDT who were prescribed or received ACTs)
Tanz1/a: 89%
Tanz1/b: 94%
Tanz1/c: 85%

Adherence to negative mRDTs
(% of patients testing negative with an mRDT who were NOT prescribed or did NOT receive any antimalarials)
Tanz1/a: 90%
Tanz1/b: 85%
Tanz1/c: 96%

Possible explanatory factors:

- **Stockouts**: there were fewer mRDT stockouts in Tanz1/c; almost half of facilities had stockouts at endline in Tanz1/a and about one quarter in Tanz1/b (where health workers also complained about running out of reagent). 56% of facilities had mRDTs in stock at the endline; about one sixth of facilities had stockouts at endline in Tanz1/c. However this does not explain all of the difference (as there was not 100% uptake when only those facilities with mRDTs in stock were included in the analysis)
- **Staffing** - in Tanz1/b some prescribers mentioned staff shortages, although it was not clear whether these affected the use of mRDTs
- **Goodness of fit with landscape of care -** adherence to negative results was lowest in Tanz1/b, where malaria prevalence was low (it was moderately high in the other two scenarios). Interviewees in Tanz1/b felt it was a challenge that so many tests were negative. This let some to mistrust the tests. Some health workers in Tanz1/a and Tanz1/b didn't trust the test, however this was not universal. Perceptions of health workers in Tanz1/c was not known.
  Some interviewees in Tanz1/b stated that they gave antimalarials to those testing negative if they had malarial symptoms, if they couldn't

find an alternative diagnosis or if they returned 2-3 days later with the same symptoms.

Related publications

1. Bruxvoort K, Kalolella A, Nchimbi H, Festo C, Taylor M, Thomson R, et al. Getting antimalarials on target: impact of national roll-out of malaria rapid diagnostic tests on health facility treatment in three regions of Tanzania. *Tropical Medicine & International Health*. 2013;18(10):1269 - 82.

Tanz2: Targeting ACT drugs: the TACT trial

Location	Northeast Tanzania
Sector targeted	Public
Intervention dates	Tanz2/1: October 2008 – June 2009<br>Tanz2/2: January 2011 – March 2012<br>Tanz2/3: January 2011 – March 2012<br>Tanz2/4: January 2011 – March 2012
Timing of evaluation	Tanz2/1: unclear<br>Tanz2/2: January 2011 – March 2012<br>Tanz2/3: January 2011 – March 2012<br>Tanz2/4: January 2011 – March 2012
Prescriber sample	Tanz2/1a: 10 facilities<br>Tanz2/1b: 10 facilities<br>Tanz2/2: 12 facilities<br>Tanz2/3: 12 facilities<br>Tanz2/4: 12 facilities
Patient sample	Tanz2/1a: 3,199<br>Tanz2/1b: 4,038<br>Tanz2/2: 9,297<br>Tanz2/3: 9,825<br>Tanz2/4: 7,963
Qualitative data collected from prescribers?	Yes

Cases:

- Tanz2/a1: Pilot intervention, Hai district in Kilimanjaro, low-moderate malaria transmission
- Tanz2/b1: Pilot intervention, Handeni district in Tanga, moderate malaria transmission
- Tanz2/2: comparison arm
- Tanz2/3: intervention arm 1
- Tanz2/4: intervention arm 2

Background context:

Tanz2/1
No facility had experience of mRDTs or microscopy. Antimalarial drugs and mRDTs were supposed to be free for children under 5 years, pregnant women and the elderly, although this didn't always happen in practice.

Tanz2/2, Tanz2/3, Tanz2/4

The study districts in Tanga and Kilimanjaro represented one moderate and one low transmission area respectively. Both were predominantly rural but contained one urban area. There was overdiagnosis of malaria, particularly in low transmission areas. mRDTs had been introduced in 2009/2010. At baseline health workers recognised that there had previously been overdiagnosis of malaria and felt empowered by mRDTs. However it was also seen as a source of conflict with patient expectations and had the potential to undermine clinical authority. There was patient demand for mRDTs, because of a desire to have their malaria confirmed.

Intervention:

Tanz2/1: Pilot study: mRDTs supplied with basic instruction on use.

Health workers were offered 1 day training on how to use the mRDT and read the result. Antimalarial drug use guidelines were reviewed and laminated job aides provided. mRDTs and associated supplies were made available, with continuous supplies ensured. Research assistants made monthly supervisory visits for evaluation purposes.

Tanz2/2: Comparison arm standard training plus mRDTs and supervision
Two-day, didactic, Ministry of Health (MoH) training on how to use mRDTs, including practical, as well as mRDT supplies every 4-6 weeks and six-weekly supervisory visits by the study team (to check clinic supplies and reporting). On average all (3) workers from each of the study health facilities attended the training.

Tanz2/3: Intervention arm 1: additional training, feedback & motivational SMS
In addition to the interventions in the comparison arm, staff were offered three additional 90 minute interactive training workshops, with one session repeated 6-7 months later. These covered:

- Adapting to the change in the diagnosis & management of malaria
- Practice with confidence when using mRDTs: tools to enable change in managing febrile illness
- Sustaining the change in practice

Experimentation with mRDTs was encouraged. In addition, approximately 5 months after training mobile-phone message (SMS) feedback was sent to health workers of their previous month's use of mRDTs (proportion of eligible patients who were tested) and treatment prescribed based on mRDT results (proportion of patients with a negative test treated with an antimalarial drug). Health workers were also sent SMS twice a day for 15 days, with a motivating message on malaria case management alternated with a motivational proverb.

Tanz2/4: Intervention arm 2: intervention arm 1 plus posters and patient leaflets
In addition to the interventions detailed in intervention arm 1, facilities were provided with posters and patient leaflets.

**Medical supply mechanism**	mRDTs supplied study, ACTs through standard mechanisms
**Were continuous supplies assured?**	Tanz2/1: mRDT supplies assured<br>Tanz2/2-4: No
**Cost of mRDTs/ACTs to patients**	Tanz2/1: not free<br>Tanz2/2-4: free mRDTs, unknown for ACTs
**Who conducted mRDT?**	Prescriber

<u>Study design</u>

Tanz2/1

Observational study. Facilities were selected for inclusion on the basis of reasonable access (within 1 hour car journey) and being a MoH-approved primary care facility. All patients attending health facility were included in the study. Data was collected from routine facility data records. Exit surveys were conducted with patients two days per week, for four months. It was not stated whether all patients were surveyed or not. The facility data was used in this analysis.

Tanz2/2-4

3-arm cluster randomised trial. Primary care dispensaries were eligible for inclusion in the study if they were in receipt of supplies of recommended antimalarial drugs from the MoH, agreed to exclusive use of mRDT for routine diagnosis of first consultations for possible malaria, were accessible by 4-wheel drive throughout the year and data were available on % consultations diagnosed with malaria in 2008 or sooner and treated more than 500 patients for malaria. All patients consulting with a new episode of a non-severe illness

were eligible for inclusion; data collected from exit interviews two days a week. Exit surveys started 3-6 months prior to the start of the intervention. Data on mRDT use and results were collected from routine dispensary records. Periods of stockout were excluded from the analysis.

Findings:

Uptake (% of patients with fever or history of fever in past 24 hours who were tested with an mRDT):
Tanz2/2: 45%
Tanz2/3: 50%
Tanz2/4: 59%

Adherence to positive mRDTs (% of patients testing positive with an mRDT who received ACTs):
Tanz2/2: 79%
Tanz2/3: 81%
Tanz2/4: 77%

Adherence to negative mRDTs (% of patients testing negative with an mRDT who did NOT receive any antimalarials):
Tanz2/1a: 77%
Tanz2/1b: 90%
Tanz2/2: 77%
Tanz2/3: 92%
Tanz2/4: 95%

Possible explanatory factors:

- **Familiarity with testing:** Interviewees in Tanz2/4 commented that they spent a lot of time educating patients; they also reported that patient demand for testing existed before mRDTs. At baseline, patients wanted to be tested. Acceptability grew with time.
- **Intervention messages:** One interviewee in Tanz2/2 seemed to misunderstand the guidelines or the training as they said they only tested if the patient had had a fever for several days (as if fever is recent, the test won't be positive); they also reported that the training said to diagnose as 'unconfirmed malaria' if an mRDT was negative but the patient had all the signs and symptoms of malaria.
  Health workers at baseline explained that the IMCI guidelines said to treat fever with antimalarials. In Tanz2/3 some said there were

exceptions to not prescribing antimalarials to mRDT negatives (e.g. if from far and with no other symptoms, or if under 5 years with fever)

- **Staffing:** One interviewee in Tanz2/3 suggested that if they were understaffed and overwhelmed with patients they would not test
- **Goodness of fit with landscape of care:** Mixed opinions on whether to trust mRDT results; some said trust came with time/experience (e.g. when they saw a patient who tested negative recover)
  Some still had doubts e.g. if they don't get many positive results, or because it only tests for one species.
  Health workers in all cases reported increasing patient acceptance over time of non-prescription of antimalarials when tested negative, whereas those at baseline they had reported a lack of acceptance.

Related publications

1. Chandler, C. I., J. Meta, C. Ponzo, F. Nasuwa, J. Kessy, H. Mbakilwa, A. Haaland and H. Reyburn (2014). "The development of effective behaviour change interventions to support the use of malaria rapid diagnostic tests by Tanzanian clinicians." Implement Sci **9**: 83.
2. Cundill, B., H. Mbakilwa, C. I. Chandler, G. Mtove, F. Mtei, A. Willetts, E. Foster, F. Muro, R. Mwinyishehe, R. Mandike, R. Olomi, C. J. Whitty and H. Reyburn (2015). "Prescriber and patient-oriented behavioural interventions to improve use of malaria rapid diagnostic tests in Tanzania: facility-based cluster randomised trial." BMC Med **13**(1): 118.
3. Leurent, B., Reyburn H, Muro F, Mbakilwa H, Schellenberg D. (2016). "Monitoring patient care through health facility exit interviews: an assessment of the Hawthorne effect in a trial of adherence to malaria treatment guidelines in Tanzania." BMC Infectious Diseases **16**: 59.
4. Hutchinson, E., Reyburn, H., Hamlyn, E., Long, K., Meta, J., Mbakilwa, H., et al. (2015). Bringing the state into the clinic? Incorporating the rapid diagnostic test for malaria into routine practice in Tanzanian primary healthcare facilities. *Glob Public Health*, 1-15.

Tanz3: Trusting rapid diagnostic tests in Zanzibar

Location	Zanzibar
Sector targeted	Public
Intervention dates	May – July 2010
Timing of evaluation	May – July 2010
Prescriber sample	12 facilities
Patient sample	3,887
Qualitative data collected from prescribers?	Yes, though after the study had been completed (6 study prescribers and 6 other prescribers interviewed in a similar study)

Background context

Study took place in two rural districts. Zanzibar's national treatment guidelines from 2009 indicate treatment with antimalarials only upon positive diagnostic test result. mRDTs were scaled up in 2006 (and introduced in some sites two years earlier, in 2004, by MSF). IMCI was revised to include mRDTs in 2009. An earlier study reported high confidence in mRDT results among health workers and patients, as well as acceptance of antimalarials only being prescribed to those with a malarial diagnosis. The authority of the health system was reported to be strong among both health workers and patients.

Intervention

Prescribers received 6-11 days IMCI training (depending on whether refresher training or for new health workers), plus one week study-specific training (including good clinical practice, provision of informed consent, performance and interpretation of mRDT according to the manufacturer's instructions).

**Medical supply mechanism**	mRDTs and ACTs supplied by MoH, with study back up
**Were continuous supplies assured?**	Yes
**Cost of mRDTs/ACTs to patients**	Free
**Who conducted mRDT?**	Prescriber

Study design

Observational study. Uptake of mRDTs was not assessed.

Five primary health care units and one primary health care centre in each of the two study districts were selected purposively. Facilities were selected to ensure adequate manpower capacity, with at least 2 health workers available per study site during the trial and a balanced geographical distribution. Prescribers were recruited to the study and paid a salary supplement for participating.

Data was collected through prescriber-completed, project specific case record forms. Patients were recruited Mon-Fri 8am to 4pm by the study health worker on duty. Patients were eligible to be included in the study if they were aged 2 months or over and presented at the study sites with fever i.e. 37.5 degrees or higher or history of fever during the preceding 24hrs, and were willing to consent to participate. Patients were excluded and referred in case of any symptoms of severe disease or danger signs. Pregnant women testing positive for malaria were excluded from the current analysis for comparability purposes.

Healthcare workers views about mRDTs were explored through interviews in a later, related qualitative study. This included six study healthcare workers and six other healthcare workers, who had not been involved in the study but had comparable mRDT experiences.

Findings

100% of patients with positive mRDTs were prescribed ACTs
100% of patients with negative mRDTs were *not* prescribed antimalarials

Possible explanatory factors:

- **High project control** – study staff (health workers) were paid extra to participate in study; there was daily contact with project team, the study team ensured a continuous supply of mRDTs, ACTs and other medicines, the study only ran (i.e. patients only enrolled) during weekday daytimes.
- **Intervention messaging**: adherence to test results was congruent with Ministry of Health malaria and IMCI guidelines.
- **Good fit with landscape of care** – interviewees were aware that malaria prevalence had declined, as was the general population.
  - health workers had had experience of mRDTs for several years. Trust in mRDTs was high; there was acceptance that not all fever was

malaria. There was a culture of high adherence in Zanzibar in general, with acceptance of Ministry of Health guidelines and interventions. Health workers didn’t seem to rely on clinical diagnosis. Some facilities had tests for other diseases e.g. urinanalysis.

- **Acceptability of alternative treatments for mRDT negative patients:** Health workers didn’t seem to see malaria as a risk, possibly due to low malaria prevalence.
- **Familiarity with testing:** Malaria messaging was widespread in the community; there was high awareness that malaria had decline and previous interventions had been successful. Patients accepted the need for testing prior to treatment. Patient acceptance of adherence to test results even at baseline; medication (antimalarials, antibiotics, antipyretics) was free for all study participants.
- **Motivation to perform well in the intervention:** prescribers were recruited to participate in the study and paid a salary supplement for the work. Other malaria research activities have been conducted in the study area in recent years – the majority of health workers interviewed had participated in past research studies; the success of previous interventions increased trust in current intervention.

Related publications

1. Baltzell K, Elfving K, Shakely D, Ali AS, Msellem M, Gulati S, et al. Febrile illness management in children under five years of age: a qualitative pilot study on primary health care workers' practices in Zanzibar. *Malaria Journal*. 2013;12:37.
2. Shakely D, Elfving K, Aydin-Schmidt B, Msellem MI, Morris U, Omar R, et al. The usefulness of rapid diagnostic tests in the new context of low malaria transmission in Zanzibar. *PLoS One*. 2013;8(9):e72912.

Uga1: The PRIME trial: Improving health centres to reduce childhood malaria in Uganda

Location	Tororo, eastern Uganda
Sector targeted	Government primary care
Intervention dates	May 2011 – Apr 2013
Timing of evaluation	July 2011 – Apr 2013
Prescriber sample	10 facilities
Patient sample	81,682
Based on formative research?	Yes, a lot
Qualitative data collected from prescribers?	Yes

Background context

A very rural area with limited infrastructure and very high malaria transmission. Many of the health facilities lacked water and electricity and often had staff shortages and issues of staff turnover. Prior to the intervention, malaria was generally diagnosed clinically, even where microscopy was available (which was only functional in a couple of study facilities). Delivery of supplies, including ACTs, had typically been unpredictable.

mRDTs were introduced nationally at the same time as the study and reached the study site 18 months after the intervention started, with training in November/December 2012. There was a general demand for and acceptance of the idea of testing (not specific to malaria) among prescribers and the public prior to the intervention. However there was also high patient demand for antimalarials. There was a paternalistic and authoritative culture of care.

Intervention

1. Training health workers in fever case management (FCM) and the use of mRDTs
   This involved a two day training session followed a week later by on-site training in facilities. Training was interactive and included performing and reading an mRDT (including practical), management of a patient with fever and either positive or negative mRDT as well as patient communication. All health workers were invited to attend the training.

2. Training health care workers on patient-centred services, including role plays on how to deal with ‘difficult’ patients including those negative for malaria one half-day session per week for six weeks
3. Training in-charges in health centre management including how to requisition and account for mRDTs and ACTs using a new system, and how to use the register records to monitor mRDT and ACT use, one half-day session per week for three weeks
4. Ensuring supplies of mRDTs and ACTs

Supervision on FCM only, with feedback, was conducted six weeks and six months after the training. The study team visited facilities initially monthly, then quarterly, for evaluation purposes.

**Medical supply mechanism**	mRDTs & ACTs supplied by government, with study back-up supply in case of stock-outs
**Were continuous supplies assured?**	Yes, both RCTs and ACTs
**Cost of mRDTs/ACTs to patients**	Free
**Who conducted mRDT?**	Prescriber

Study design

Two-arm cluster randomised controlled trial, with the control arm receiving no additional training or assured supplies (not included in current analysis). Outcome data was collected through cross-sectional community surveys, a cohort study, facility registers completed by prescribers and exit interviews with caregivers of under 5 year olds. The registers were used for the current analysis.

All prescribers were invited to participate in the intervention. All patients visiting facilities were included in the study.

Findings

According to facility registers, 98% of patients with history of fever in past 48 hours were tested with an mRDT. However these findings differed from other sources of data such as patient exit interviews. Table 1 below shows that the majority of patients seen did not have their fever status recorded and that most of those for whom fever status was recorded, had a fever or history of fever. This suggests that there was recording bias in the registers, which likely overestimate the proportion tested.

Table 1: Variation in mRDT uptake by data source, denominator and age group

mRDT uptake	All ages	U5s
% patients with recorded fever/history of fever, tested with an mRDT according to facility registers (n)[1]	98% (49,778/50,615)	99% (19,317/19,537)
% all patients tested with an mRDT according to facility registers (n)[2]	36% (49,778/139,465)	44% (19,317/43,804)
% patients with a reported fever/history of fever, tested with an mRDT according to exit interview (n)	n/a	68% (475/696)
% all patients tested with an mRDT according to exit interview (n)	n/a	d/k

[1] Note, fewer than half of patients recorded in registers had their fever status recorded. This was a new section of the register, along with the mRDT information, and may only have been completed for patients who went on to be tested. Of those with fever status recorded, 89% of all patients and 95% of under 5 patients were recorded as febrile.

[2] Of children under 5 presenting to health facilities in this area, we can infer from other data that at least 90% will be febrile. This suggests that mRDT uptake is substantially lower in practice than when defined by those recorded as febrile.

According to facility registers, 93% of patients of all ages testing positive with an mRDT were prescribed or received ACTs and 3% of patients testing negative with an mRDT were prescribed or received antimalarials. However according to exit interviews with caregivers of patients under 5 years of age, 77% of those who had a positive reference microscopy reported receiving an ACT and 31% of those who had a negative reference microscopy reported receiving antimalarials.

Possible explanatory factors:

- **Mixed motivation to perform well in the intervention** – prescribers viewed the intervention as being implemented by an external organisation, from whom many felt they should have been getting a 'motivation', i.e. a financial incentive or gift, for the additional work expected as part of the study.
- **Patient acceptability** – prescribers reported that patients were happy to have blood tests; they appreciated having a diagnosis. They reported that patient numbers increased/patients were coming to the facilities from far away. Patients' experiences of recovery increased prescribers' trust.
- **Prescriber acceptability** – prescribers reported feeling that mRDTs enhanced their practice, or helped them to do their job better. It made them proud and was not difficult to do mRDTs.

- **High coverage of malaria training -** training delivered to almost all staff, 25/28. Supervision provided on-site to trouble-shoot and re-emphasise good practice. Supervision also reached some health workers who had not attended the initial training (e.g. new staff). However, if a staff member had not been trained, they typically did not conduct mRDTs (although they may also not have been recording fever status either).
- **Workload –** prescribers explained that they did not test if the facility was understaffed, particularly if there was only one staff member in the facility, or if they were over-worked, with high patient numbers.
- **Possible data collection bias** - process reports noted some staff and one facility where mRDTs were not done; health workers interviewed explained that in circumstances of high workload and understaffing, or if new staff joined the facility who had not been trained, mRDTs may not be conducted; concerns were also raised in process reports that registers may not be completed accurately; rate of uptake lower when measured by exit interviews for under 5 year olds
- **Trust in mRDTs** - health workers seemed to trust the mRDT's accuracy, possibly due to high mRDT positivity rates.

Related publications

1. Chandler, C.I., et al., *The PROCESS study: a protocol to evaluate the implementation, mechanisms of effect and context of an intervention to enhance public health centres in Tororo, Uganda.* Implement Sci, 2013. **8**: p. 113.
2. DiLiberto, D., et al*, Behind the scenes of the PRIME intervention: Designing a complex intervention to improve malaria care at public health centres in Uganda,* Global Health Action, 8: p. 29067
3. *Staedke, S.G., Evaluating the impact of a public health centre intervention on management of malaria and health outcomes of children in Uganda – Results from the PRIME & PROCESS studies. Policy Brief*. 2014.
4. Staedke, S.G., et al., *The PRIME trial protocol: evaluating the impact of an intervention implemented in public health centres on management of malaria and health outcomes of children using a cluster-randomised design in Tororo, Uganda.* Implement Sci, 2013. **8**: p. 114.
5. Staedke, S. G., C. Maiteki-Sebuguzi, D. Diliberto, E. Webb, L. Mugenyi, E. Mbabazi, S. Gonahasa, S. P. Kigozi, B. Willey, G. Dorsey, M. R. Kamya and C. I. R. Chandler (2016). *The Impact of an Intervention to Improve Malaria Care in Public Health Centers on Health Indicators of Children in Tororo, Uganda (PRIME): A Cluster-Randomized Trial.* American Journal of Tropical Medicine & Hygiene, 2016, 95(2) pp 258 - 367.
6. Chandler, C. I. R., Webb, E. L., Maiteki-Sebuguzi, C. et al, *The impact of malaria rapid diagnostic tests on fever case management in a high transmission setting in Uganda: A mixed-methods cluster-randomized trial (PRIME).* PLOS One, forthcoming

Uga2: Use of rapid diagnostic tests to improve malaria treatment in the community in Uganda

Location	Rukungiri district, Southwest Uganda
Sector targeted	Voluntary community medicine distributors (CMDs)
Intervention dates	June 2010 – December 2011
Timing of evaluation	January – December 2011
Prescriber sample	90 CMDs in 32 villages in both cases
Patient sample	Uga2/a: 897 (low transmission case)<br>Uga2/b: 5,698 (moderate transmission case)
Qualitative data collected from prescribers?	Yes

Background context

A rural area with dispersed settlements and mountainous terrain. About half the community medicine distributors (CMDs) had previously volunteered to supply antimalarials for the home-based management of fever (HBMF) strategy, although that had ended prior to this study. There were two-three CMDs per village. They had not heard of mRDTs prior to the intervention but viewed them positively. It was recognised that non-malarial fevers were currently untreated and that mRDTs could help address this. CMDs were well integrated into the community, having long term relationships with patients who trusted them. mRDTs had been rolled out in Health Centre IIs in November 2008, although some continued to diagnose presumptively.

Cases:
Uga2/a: low-moderate transmission area
Uga2/b: moderate-high transmission area

Intervention

All CMDs were given four days interactive training, covering how to perform an mRDT (including practical), how to prescribe antimalarials, how to deal with negative cases and communication skills. They were also given pictorial job aids. Their role was to test children (3-59 months) for malaria and, if positive, give antimalarials. If and mRDT was negative, they were to refer children with specific symptoms to the health facility or else ask them to go home but return

if they had not recovered in two days. For severe cases, they could give rectal artesunate without testing, and refer. mRDTs and treatments were provided free of charge.

For the first six months of the intervention, CMDs had close supervision, which was then scaled back for the remainder of the intervention. After this, there were monthly parish meetings to collect supplies. CMDs were volunteers but received incentives such as t-shirts, bicycles and a kerosene allowance. Community sensitisation activities also took place.

**Medical supply mechanism**	mRDTs & ACTs supplied by study (collected at monthly parish meetings)
**Were continuous supplies assured?**	Yes, both RCTs and ACTs
**Cost of mRDTs/ACTs to patients**	Free
**Who conducted mRDT?**	Prescriber

Study design

Two-arm cluster randomised controlled trial. The control arm CMDs offered presumptive treatment of malaria and were not included in the current analysis. The intervention arm was separated into two cases for the current analysis: an area of low malaria transmission and an area of moderate-high transmission:

The evaluation ran for the final year of the intervention, after close supervision had ended. Data were collected from a project-specific register. Data was collected on all patients seeking treatment for fever.

Community members were asked to identify volunteers for the role of Community Medicine Distributor (CMD) at village meetings. All patients with fever consulting CMDs were included. The intervention targeted under 5s.

Findings

Uptake (% of patients who were tested with an mRDT):
Uga2/a: 97% of patients were tested with an mRDT
Uga2/b: 100% of patients were tested with an mRDT

Adherence to positive mRDTs
(% of patients testing positive with an mRDT who received ACTs):
Uga2/a: 66%
Uga2/b: 98%

Adherence to negative mRDTs
(% of patients testing negative with an mRDT who did NOT receive any antimalarials):
Uga2/a: 97%
Uga2/b: 99%

Possible explanatory factors:

- **High motivation to perform well in the intervention** – the CMD role was newly created for this intervention. CMDs reported because of their role, they gained status and respect from the community and parents.
- **Community acceptance** – there were few refusals for testing
  CMDs explained that the community appreciated that the CMDs offered a free service, so it saved them money. The CMDs were nearby, whereas health facilities were far, they were accessible at night when facilities are closed. Parents also liked testing itself because they liked to know if their child had malaria or not.
  However some CMDs explained that a few parents complained that it took time and tests were always negative in Uga2/a, so they preferred the presumptive CMDs (in the control arm of the study). At the same time in Uga2/b, some CMDs reported that children from presumptive villages (i.e. the control arm) came to them for testing. This suggests that the availability of mRDTs affected treatment seeking behaviour (see subsequent point).
- **Self-selecting sample** - the community knew that the CMD's role was to test, so presumably would not visit them unless they wanted their child to be tested (indeed there were far more consultations in the 'presumptive' control arm, particularly in Uga2/a, suggesting there may have been greater demand for antimalarials than for testing, although the population there was also larger. Visits to intervention CMDs were typically delayed longer after the onset of symptoms than in presumptive arms, suggesting that the decision to go for testing may have delayed treatment seeking.
- Some health facilities didn't test, which may have been a further reason to use CMDs.

- **mRDT/ACT supplies** - Although stock outs of mRDTs and ACTs were not expected and did not appear to be an issue, they were more likely in Uga2/a than in Uga2/b.
- **Acceptability to parents/community**: Although there were some cases when CMDs reported pressure from parents, explaining that they were forced to give antimalarials, in general there was acceptance that no antimalarials were given if mRDT results were negative. CMDs reported that acceptance came with time and experience of recovery without coartem, or no recovery in spite of coartem, for negative cases. There was a common understanding that not all fever was malaria. Not giving antimalarials to children with negative mRDT results may also have been acceptable to both CMDs and caregivers because their new, specific role meant that there was no expectation that they would treat all illnesses, or those that were not malaria. Their new, additional service may also have been accepted since it did not disrupt or prevent their usual treatment-seeking. Those wishing to use antimalarials could still have visited their normal source of treatment if they did not receive the outcome they were hoping for. Relatedly, some CMDs described situations when some caregivers weren't happy with negative mRDT results and would go to a presumptive CMD to get antimalarials if test was negative.
- **Trust in mRDTs** - CMDs generally reported that they trusted mRDT results; this trust grew with time and experience. Some CMDs in Uga2/b did not trust the test, for example when negative cases improved with coartem. Since they were embedded in the community, it was more likely that they would be able to get feedback or follow up patients than would have been the case in health facilities. Some health facilities did not use mRDTs, which may also have undermined the CMD's and community's trust in mRDT results.

Related publications

1. Lal, S., et al. (2015). "Health facility utilisation changes during the introduction of community case management of malaria in South Western Uganda: An interrupted time series approach." PLoS One **10**(9): 1371.

Uga3: Introducing rapid diagnostic tests in drug shops to improve the targeting of malaria treatment

Location	Mukono, Uganda
Sector targeted	Registered, private drug shop vendors in trading centres and urban areas
Intervention dates	October 2010 – December 2011
Timing of evaluation	January – December 2011
Prescriber sample	9 clusters containing 29 drug shops
Patient sample	8,561
Qualitative data collected from prescribers?	Yes

Background context

A mainly rural area with a peri-urban district. High prevalence of malaria. Testing was not the norm for drug shop vendors (DSVs) prior to the intervention and there was a general belief that they could diagnose without testing. However they felt that mRDTs could attract customers and improve their reputation. It was felt that the introduction could lead to a shift from antimalarial prescriber to antimalarial gatekeeper. Prior to the intervention, "mRDTs were not wholly unfamiliar to community members, and importantly that there was a pre-existing perception that not all fevers are malaria, and that diagnostic testing was viewed as potentially helpful in reducing this uncertainty" [4]. However it was believed that but that adhering to negative results would not be acceptable to patients. There was close social proximity between DSVs and their customers – they were trusted. The transactional nature of the relationship between DSVs and their customers gave the latter power in terms of negotiating treatment. Coartem was scarce and expensive prior to the project.

Intervention

Four days of interactive training were provided to all DSVs, which covered performing and reading mRDTs (including a practical), prescribing antimalarials, how to deal with mRDT negatives and communicating and negotiating with patients. New DSVs were not given supplies until they had received training. If an mRDT result was negative, DSVs were supposed to refer. Weekly support supervision with feedback was provided for the first two months after training.

The community were sensitised about mRDTs through leaflets distributed by village health teams and roadside placards advertised the availability of testing

at drug shops; some interviewees also mentioned megaphones advertising new testing services.

**Medical supply mechanism**	mRDTs & ACTs supplied by study (collected from study office)
**Were continuous supplies assured?**	Yes, both RCTs and ACTs
**Cost of mRDTs/ACTs to patients**	Subsidised
**Who conducted mRDT?**	Prescriber

Study design

Two-arm cluster randomised controlled trial. The control arm received no mRDTs and was not included in the current analysis. Evaluation ran for the final 12 months of the intervention, after close supervision had ended. Patients were eligible if they had a fever or history of fever when presenting at the drug shop. Data was collected from a project-specific register completed by the drug shop workers.

Findings

Uptake
(% of patients with history of fever in past 48 hours who were tested with an mRDT):
99%

Adherence to positive mRDT results:
(% of patients testing positive with an mRDT who received ACTs):
98%

Adherence to negative mRDT results:
(% of patients testing negative with an mRDT who did NOT receive any antimalarials):
99%

Possible explanatory factors:

- **High motivation to perform well in the intervention** – the intervention enhanced DSVs' status, increasing their perceived legitimacy and professionalism by giving them training, allowing them to test blood and providing visible interactions with the Ministry of Health. DSVs reported that it was good for their business, with more customers visiting their drug shop. Receiving mRDTs and coartem free from the study, which they sold

to customers, also boosted their income further. DSVs also reported that mRDTs improved the care they could offer, that they gained confidence in treating patients and that they simplified their work. They also reported finding mRDTs easy to use.

- **Data collection -** DSVs explained that they were able to sell non-project antimalarials, presumably if a patient demanded them in spite of an mRDT negative result, or if a patient refused to test. These would not have been captured in the project register (and so could have led to bias in the data collection).
- **Fitted well into landscape of care** – although very different from the existing process, prescribers were happy to integrate mRDTs into their consultations. They also reported that patients were happy to be tested, with few refusals. DSVs explained that patient acceptance came with time, but that in general, they liked blood testing per se and they liked to know their diagnosis. There was a common understanding that not all fever was malaria and so they understood needed to test before treatment. Patient acceptability could also be seen by the fact that DSVs reported that the number of patients seen increased, with word of mouth encouraging others to attend. Patients felt that the drug shops were not just selling medicines but providing a service. mRDTs increased their trust and confidence in the DSVs, who were then seen as legitimate part of the health service ('real health workers').

Related publications

1. Mbonye, A. K., et al. (2015). "A Cluster Randomised Trial Introducing Rapid Diagnostic Tests into Registered Drug Shops in Uganda: Impact on Appropriate Treatment of Malaria." PLoS One **10**(7): e0129545.
2. Mbonye AK, Ndyomugyenyi R, Turinde A, Magnussen P, Clarke S, Chandler C. The feasibility of introducing rapid diagnostic tests for malaria in drug shops in Uganda. *Malaria Journal*. 2010;9:367.
3. Chandler CI, Hall-Clifford R, Turinde A, Magnussen P, Clarke S, Mbonye AK. Introducing malaria rapid diagnostic tests at registered drug shops in Uganda: Limitations of diagnostic testing in the reality of diagnosis. *Social Science and Medicine*. 2011;72(6):937 - 44.
4. Mbonye AK, Lal S, Cundill B, Hansen KS, Clarke S, Magnussen P. Treatment of fevers prior to introducing rapid diagnostic tests for malaria in registered drug shops in Uganda. *Malaria Journal*. 2013;12:131.
5. Mbonye AK, Magnussen P, Chandler CI, Hansen KS, Lal S, Cundill B, et al. Introducing rapid diagnostic tests for malaria into drug shops in Uganda: design and implementation of a cluster randomized trial. *Trials*. 2014;15:303.
6. Hutchinson E, Chandler C, Clarke S, Lal S, Magnussen P, Kayendeke M, et al. 'It puts life in us and we feel big': shifts in the local health care system during the introduction of rapid diagnostic tests for malaria into drug shops in Uganda. *Critical Public Health*. 2015;25(1):48 - 32.
