# Supplementary material for: Improving prescribing practices with rapid diagnostic tests (RDTs): synthesis of 10 studies to explore reasons for variation in malaria RDT uptake and adherence
Source: BMJ Open. 2017 Mar 8;7(3):e012973. doi: 10.1136/bmjopen-2016-012973 (PMC5353269; doi:10.1136/bmjopen-2016-012973)
Supplement: supplementary file [file bmjopen-2016-012973supp2.pdf]

TRIAL MODE - a valid license will remove this message. See the keywords property of this PDF for more information.

Supplementary file 2: Sample of data extraction table for ‘adherence to negative mRDT results’: selected characteristics only

Case	No AM to negative RDT %	Intervention content	Duration of RDT/ malaria-focused training	Training style for RDT/ malaria training	Training on how to treat patients with negative RDT results?	Training covered skills around communicating with patients?	Support supervision	Additional intervention components
Nig1/a3	27	Enhanced	2	Interactive	Yes, though minimal	Yes	Yes - monthly, with feedback, and telephone support	Yes (school-based activities)
Cam1/a1	47	Basic	1	Didactic	Yes	No	Yes - monthly, with feedback	No
Cam1/b1	49	Basic	1	Didactic	Yes	No	Yes - monthly, with feedback	No
Ghan1/b	51	Basic	2		Yes	No	No	No
Ghan1/a	54	Basic	2		Yes	No	No	No
Nig1/a1	56	Basic	0.5	Didactic	No	No	No	No
Nig1/b1	57	Basic	0.5	Didactic	No	No	No	No
Nig1/a2	65	Enhanced	2	Interactive	Yes, though minimal	Yes	Yes - monthly, with feedback, and telephone support	No
Afgh1/c	65	Basic	1.5	Didactic	No	No	No	No
Nig1/b2	70	Enhanced	2	Interactive	Yes, though minimal	Yes	Yes - monthly, with feedback, and telephone support	No
Cam1/a2	76	Enhanced	3	Interactive	Yes	Yes	Yes - monthly, with feedback	No
Cam1/b2	77	Enhanced	3	Interactive	Yes	Yes	Yes - monthly, with feedback	No
Tanz2/a1	77	Basic	1	Didactic	No	No	No	No
Tanz2/2	78	Basic	2	Didactic	No	No	No	No
Afgh1/b	81	Basic	1.5	Didactic	No	No	No	No
Nig1/b3	81	Enhanced	2	Interactive	Yes, though minimal	Yes	Yes - monthly, with feedback on performance, and telephone support	Yes (school-based activities)
Tanz1/b	85	Basic	2	Didactic	No	No	Yes - 32% had received supervision on use of RDTs; 29% on ACTs	No

*Intervention explicitly designed to address and support the process of change in implementing RDTs, beyond knowledge and equipment, e.g. training content and methods reflected how new ideas and demands will work in practice, e.g. reflection-feedback opportunities, multiple sessions of training, role plays and feedback.

TRIAL MODE - a valid license will remove this message. See the keywords property of this PDF for more information.

Case	No AM to negative RDT %	Intervention content	Duration of RDT/ malaria-focused training	Training style for RDT/ malaria training	Training on how to treat patients with negative RDT results?	Training covered skills around communicating with patients?	Support supervision	Additional intervention components
Afgh1/a	87	Basic	1.5	Didactic	No	No	No	No
Tanz2/b1	90	Basic	1	Didactic	No	No	No	No
Tanz1/a	90	Basic	2	Didactic	No	No	Yes - 26% had received supervision on use of RDTs; 31% on ACTs	No
Tanz2/3	92	Enhanced	3	Interactive	Yes	Yes	?	Yes (feedback & motivational text messages)
Tanz2/4	95	Enhanced	3	Interactive	Yes	Yes	?	Yes (feedback & motivational text messages; patient leaflets)
Tanz1/c	96	Basic	2	Didactic	No	No	Yes - 22% had received supervision on use of RDTs; 19% on ACTs	No
Uga1	97	Enhanced	3	Didactic	Yes	Yes	Yes – at one week, 6 weeks and 6 months, with feedback	Yes (training on patient-centred services, training in-charges in health centre management)
Uga2/a	97	Enhanced	4	Interactive	Yes, though minimal	Yes	Yes - close supervision for first 6 months (before evaluation)	Yes (community sensitisation)
Uga2/b	99	Enhanced	4	Interactive	Yes, though minimal	Yes	Yes - close supervision for first 6 months (before evaluation)	Yes (community sensitisation)
Uga3	99	Enhanced	4	Interactive	Yes, though minimal	Yes	Yes - weekly, with feedback, for first 2 months (before evaluation)	Yes (community sensitisation)
Tanz3	100	Enhanced	d/k (6-11 for all IMCI training, not just malaria)	Interactive	Yes	Yes	Yes	Yes (IMCI training, salary for providers)

*Intervention explicitly designed to address and support the process of change in implementing RDTs, beyond knowledge and equipment, e.g. training content and methods reflected how new ideas and demands will work in practice, e.g. reflection-feedback opportunities, multiple sessions of training, role plays and feedback.

TRIAL MODE - a valid license will remove this message. See the keywords property of this PDF for more information.

*Intervention explicitly designed to address and support the process of change in implementing RDTs, beyond knowledge and equipment, e.g. training content and methods reflected how new ideas and demands will work in practice, e.g. reflection-feedback opportunities, multiple sessions of training, role plays and feedback.
